# Modern Approach to Testing the Biocompatibility of Osteochondral Scaffolds in Accordance with the 3Rs Principle—Preclinical *In Vitro*, *Ex Vivo*, and *In Vivo* Studies Using the Biphasic Curdlan-Based Biomaterial

**DOI:** 10.1021/acsbiomaterials.4c01107

**Published:** 2025-01-20

**Authors:** Katarzyna Klimek, Sylwia Terpilowska, Agnieszka Michalak, Rafal Bernacki, Aleksandra Nurzynska, Magali Cucchiarini, Marta Tarczynska, Krzysztof Gaweda, Stanisław Głuszek, Grazyna Ginalska

**Affiliations:** † Chair and Department of Biochemistry and Biotechnology, Medical University of Lublin, Chodzki 1 Street, 20-093 Lublin, Poland; ‡ Department of Surgical Medicine with the Laboratory of Medical Genetics, Jan Kochanowski University, Collegium Medicum, IX Wiekow Kielc 19A Av., 25-317 Kielce, Poland; § Independent Laboratory of Behavioral Studies, Medical University of Lublin, Chodzki 4a Street, 20-093 Lublin, Poland; ∥ Veterinary Clinic Aura, Debowa 31 Street, 86-065 Lochowo, Poland; ⊥ Center of Experimental Orthopaedics, Saarland University Medical Center, Saarland University, Kirrbergerstr. Bldg 37, 66421 Homburg/Saar, Germany; # Department and Clinic of Orthopaedics and Traumatology, Medical University of Lublin, Jaczewskiego 8 Street, 20-954 Lublin, Poland; ¶ Faculty of Health Sciences, Vincent Pol University, Choiny 2 Street, 20-816 Lublin, Poland

**Keywords:** osteochondral scaffolds, cytotoxicity, genotoxicity, inflammation, cytocompatibility in vitro, biocompatibility in vivo

## Abstract

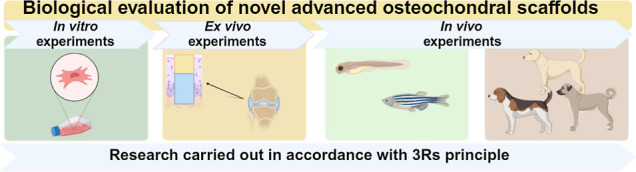

The aim of this work is to provide a comprehensive set of biological tests to assess the biomedical potential of novel osteochondral scaffolds with methods proposed to comply with the 3Rs principle, focusing here on a biphasic Curdlan-based osteochondral scaffold as a promising model biomaterial. *In vitro* experiments include the evaluation of cytotoxicity, mutagenicity, and genotoxicity referring to ISO standards, the assessment of the viability and proliferation of human chondrocytes and osteoblasts, and the estimation of inflammation after direct contact of biomaterials with human macrophages. *Ex vivo* experiments include assessments of the response of the surrounding osteochondral tissue after incubation with the implanted biomaterial. *In vivo* experiments involve an evaluation of the toxicity and regenerative potential of the biomaterial in zebrafish (larvae and adults) and in osteochondral defects in dogs (veterinary patients). The applied set of tests allows us to show that the Curdlan-based scaffold does not induce cytotoxicity (cell viability close to 100%), mutagenicity (the level of reversion is not 2× higher compared to the control), and genotoxicity (it does not exhibit any change in chromosomal aberration; the frequency of micronuclei, micronucleated binucleated cells, and cytokinesis-block proliferation index is comparable to the control; moreover, it does not cause the formation of comets in cells). This biomaterial also promotes the viability and proliferation of chondrocytes and osteoblasts (the OD values between the fourth and seventh day of incubation increase by approximately 1.6×). The Curdlan-based scaffold stimulates only a transient inflammatory response *in vitro* and *ex vivo*. This biomaterial does not cause *Danio rerio* larvae malformation and also enables proper regeneration of the caudal fin in adults. Finally, it supports the regeneration of an osteochondral defect in veterinary patients. Thus, this is a proposal to use alternative methods for biological assessment of osteochondral scaffolds as opposed to commonly used tests using large numbers of laboratory animals.

## 1 Introduction

Cartilage damage resulting from injuries during training, diseases, or pathological factors can lead to a significant loss of patients’ mobility and quality of life. The most serious and extensive cartilage lesions (osteochondral—OC—defects, Outerbridge and International Cartilage Repair Society grade IV) are a huge therapeutic challenge because they include not only cartilage but also the underlying subchondral bone.^1,2^ To date, many therapeutic methods such as arthroscopic debridement, microfracture, or mosaicplasty using autografts or allografts have been developed.^3−10^ Nevertheless, all the mentioned therapies have certain advantages and limitations, and therefore, increasing attention is being paid to the use of artificial bioactive OC scaffolds. Thus, scientists around the world are constantly developing new OC biomaterials that will replace the damaged OC tissue and enable its complete regeneration.^11−16^

Desirable OC scaffolds must, therefore, meet many requirements. First, they should be biomimetic, i.e., their composition, structure, and properties should imitate the natural OC tissue. Moreover, OC scaffolds should be nontoxic, and they also should support cell adhesion, proliferation, and differentiation as well as tissue ingrowth. On the other hand, such biomaterials should possess mechanical properties similar to the native OC tissue. Therefore, due to the complexity of the OC tissue, biphasic, triphasic, and multiphasic scaffolds are produced using natural and synthetic polymers as well as ceramics.^17−25^

Typically, newly developed OC scaffolds are subjected to structural (e.g., scanning electron microscopy analysis), physicochemical (e.g., Fourier transform infrared analysis or X-ray diffraction analysis), and mechanical tests. Then, these biomaterials are subjected to biological experiments, including evaluation of cell response *in vitro* (often very preliminary), and then they are immediately intended for implantation in the OC defects of mice, rats, rabbits, or even larger laboratory animals such as dogs or sheep.^26−29^ It should be emphasized that while *in vivo* studies using the above-mentioned laboratory animals allow for the assessment of biomaterial’s biocompatibility, they are costly and, second, require the involvement of a huge number of laboratory animals to evaluate a few variants of scaffolds and to obtain statistically significant results. Moreover, according to the 3Rs principle (replacement, reduction, refinement), biological research should primarily include several alternative analyses that avoid the use of animals in research. Moreover, even though research in animal models is necessary, those with the lowest possible pain threshold should be used, and their numbers should be reduced to a minimum. These studies should also be carried out according to improved procedures aiming at providing animals with the best possible experimental conditions (with maintaining and improving their welfare).^30,31^

Taking into account such appreciation, the aim of this work was to provide alternative methods for assessing the biomedical potential of OC biomaterials in accordance with the 3Rs principle. To do so, a biphasic OC scaffold based on Curdlan was used as a model biomaterial. This biomaterial has been previously structurally, physicochemically, and mechanically characterized, and its preliminary cytocompatibility *in vitro* has been assessed.^32^ Thus, initial studies have shown that it has a biomimetic nature with a compact structure (no delamination at the interface was noticed) and also supports the growth, proliferation, and differentiation of human mesenchymal stem cells *in vitro*. In this study, such a biomaterial was subjected to additional alternative biological tests (*in vitro*, *ex vivo*, and *in vivo* studies) in order to comprehensively evaluate its potential as an OC scaffold. Therefore, its cytotoxicity was assessed according to the ISO 10993-5:2009 standard “Biological evaluation of medical devices—Part 5: Tests for *in vitro* cytotoxicity”,^33^ while its mutagenicity and genotoxicity were evaluated according to the ISO 10993-3:2014 standard “Biological evaluation of medical devices—Part 3: Tests for genotoxicity, carcinogenicity and reproductive toxicity”.^34^ These experiments were performed using the recommended CHO-K1 cell line (Chinese hamster cells), as it is characterized, among other things, by good growth and stable karyotype.^34^ Moreover, the response of human chondrocytes, osteoblasts, and macrophages in direct contact with the biomaterial *in vitro* was monitored. *Ex vivo* experiments were conducted using well-established and accepted experimental OC defects created in human cartilage biopsies to analyze its behavior in a more natural environment.^35^ In turn, *in vivo* studies were performed using zebrafish (*Danio rerio*). Therefore, the effect of the biomaterial on the development of cartilage in zebrafish larvae and on the regeneration of the amputated caudal fin in adults was investigated. The biomaterial was also implanted in three veterinary patients (dogs) suffering from *Osteochondritis Dissecans* (OCD). We hypothesize that the conducted experiments will allow us to determine the biomedical potential of the aforementioned biphasic Curdlan-based biomaterial in accordance with the 3Rs principle. The presented research constitutes a modern and alternative approach to assess the biocompatibility of OC scaffolds, and we assume that it may be a useful set of tests allowing for the selection of the most promising variant of the biomaterial before conducting tests on laboratory animals.

## 2 Materials and Methods

### 2.1 Materials

Curdlan powder from *Alcaligenes faecalis* var. *myxogenes* (cat. no. 281-80531) was supplied by FUJIFILM Wako Pure Chemical Corporation (Hong Kong, Japan). Whey protein isolate (WPI)—BiPRO 9500 was provided by Agropur Cooperative, USA. Hydroxyapatite (HAp) granules were produced themselves according to our procedure described in detail previously.^36^ The CHO-K1 cell line (cat. no. CCL-61), hFOB 1.19 cell line (cat. no. CRL-11372), and THP-1 cell line (cat. no. TIB-202) were purchased from American Type Culture Collection (ATCC), USA. The F-12 K medium (cat. no. 30-2004), RPMI-1640 medium (cat. no. 30-2001), Dulbecco’s modified Eagle’s medium (DMEM)/F12 1:1 medium (cat. no. 11330032 for chondrocyte culture and cat. no. 11039021 for osteoblast culture) were supplied by ATCC, USA, and Gibco Thermo Fisher Scientific, USA, respectively. Fetal bovine serum (FBS Good, cat. no. P40-37500) was from Pan-Biotech, Germany. Human fibroblast growth factor 2 (hFGF-2, cat. no. 233-FB-010) and human transforming growth factor β-1 (hTGF-β1, cat. no. 240-B-002) were provided by R&D SYSTEMS, USA. Oxy Select 96-well Comet Assay Slides (cat. no. STA-356) were purchased from Cell Biolabs Inc., USA, while Alexa-Fluor 635 Phalloidin (cat. no. A34054) from Invitrogen, Thermo Fisher Scientific, USA. The human interleukin 6 (IL-6) ELISA Kit (cat. no. D6050), human macrophage inflammatory protein 1 beta (MIP-1β) ELISA Kit (cat. no. DMD00), and human tumor necrosis factor alpha (TNF-α) ELISA Kit (cat. no. DTA00C) were supplied by R&D SYSTEMS, USA. The remaining reagents, Cell Counting Kit-8 (cat. no. 96992), lipopolysaccharides from *Escherichia coli* O111:B4 (cat. no. L4391), G418 disulfate salt solution (cat. no. G8168), cytochalasin B from *Drechslera dematioidea* (cat. no. C6762), acridine orange (cat. no. 158550), boric acid (cat. no. B6768), ethylenediaminetetraacetic acid disodium salt dehydrate (EDTA) (cat. no. E5134), trizma base primary standard and buffer (cat. no. T1503), S9 form Liver, pooled (S2442-1VL), magnesium chloride, anhydrous (cat. no. M8266), potassium chloride (cat. no. P3911), phosphate buffer solution (PBS) (cat. no. P3619), glucose-6-phosphate (cat. no. 10127647001), NADP disodium salt (cat. no. 10128031001), mitomycin C (cat. no. 10107409001), cyclophosphamide monohydrate (cat. no. C0768), potassium chloride solution (cat. no. P8327), colcemid (cat. no. 10295892001), ethanol absolute (cat. no. 1070172511), chloroform (cat. no. 366927), methanol (cat. no. 34860), sodium chloride (cat. no. S9888), Giemsa stain (cat. no. G5637), Dulbecco’s phosphate-buffered saline without calcium chloride and magnesium chloride (cat. no. D8537), penicillin–streptomycin solution (cat. no. P4333), dimethyl sulfoxide (DMSO), sterile filtered (cat. no. D2438), thiazolyl blue tetrazolium bromide (cat. no. M5655), 4-nitro-1,2-phenylendiamine (cat. no. 108898), methylmethanesulfonate (cat. no. 78697), 2-aminoanthracene (cat. no. A38800), acetic acid solution (cat. no. 45754), Live/Dead Cell Double Staining Kit (cat. no. 04511), Bis-benzimide H 33342 trihydrochloride (cat. no.14533), 2-mercaptoethanol (cat. no. M3148), phorbol 12-myristate 13-acetate (cat. no. P1585), interferon-γ human (cat. no. I3265), formaldehyde solution (cat. no. 47608), sodium citrate (cat. no. 71497), formic acid (cat. no. F0507), safranin O (cat. no. S2255), tricaine methanesulfonate (cat. no. E10521), Alcian-blue staining solution (cat. no. TMS-010-C), glycerol (cat. no. G5516), and potassium hydroxide (cat. no. 221473), were provided by Merck SA (Darmstadt, Germany).

### 2.2 Preparation of the Osteochondral Scaffold

The Curdlan-based biomaterial was fabricated according to Polish Patent no. 240639 as described in detail previously.^32^ The fabrication procedure of the scaffold can be divided into the following steps: (I) preparation of the polymer mixture; (II) deposition of HAp granules in the lower part of the polymer mixture via sedimentation, followed by centrifugation; (III) gelation of the composite by thermal incubation; and (IV) sterilization. First, 0.08 g of Curdlan powder was suspended in 1 mL of 30% w/v WPI solution. The mixture was prepared in a 2 mL Eppendorf tube. Then, HAp granules were added to the obtained solution. The polymer–ceramic mixture was subjected to a sedimentation process and additionally centrifuged (Centrifuge MiniSpin plus, Eppendorf, Poland) to obtain two phases, Curdlan/WPI top phase and Curdlan/WPI/HAp bottom phase. Then, the biomaterial was cross-linked by incubation at 90 °C for 15 min (Fixed Dry Block Heater, BTF, Grant Instruments, USA). This procedure was repeated until the appropriate amount of biomaterials was fabricated. For experiments with the use of liquid extracts, *ex vivo* studies, and implantation procedures, biomaterials with 10 mm height (top layer with approximately 3 mm height and bottom layer with approximately 7 mm height) were prepared. In turn, for cell culture experiments in direct contact with biomaterials, cylinder-shaped samples with approximately 3 mm height were made. The biomaterials were sterilized by autoclaving (121 °C, 20 min, Prestige Medical Classic Media, Prestige Medical, UK).

### 2.3 Evaluation of Cytotoxicity *In Vitro*

The cytotoxicity was assessed according to the ISO 10093-5:2009 standard,^33^ while the liquid extracts were prepared according to the ISO 10093-12:2012 standard.^37^ The experiment was performed toward the Chinese hamster cells—CHO-K1 cell line (model cell line recommended by ISO standards).^33,34^ Cells were seeded in 96-well plates at a concentration of 3 × 10^4^ cells/well and incubated for 24 h (37 °C, 5% CO_2_, Heraeus cytoperm 2 CO_2_ 6-Door Incubator, Thermo Fisher Scientific, USA). In order to prepare liquid extracts, the biomaterials were incubated in the culture medium (F-12K Medium, 10% FBS, 100 U/mL penicillin, and 100 μg/mL streptomycin) in the proportion of 100 mg of biomaterial per 1 mL of medium (24 h, 37 °C, Heraeus cytoperm 2 CO_2_ 6-Door Incubator, Thermo Fisher Scientific, USA). In parallel, a control extract was prepared by incubating the culture medium without biomaterials (negative control of cytotoxicity). In turn, a 1% DMSO solution was used as a positive control of cytotoxicity. After 24, 48, and 72 h of incubation, cell viability was evaluated using the MTT test—[3-(4,5-dimethylthiazol-2-yl)-2,5-diphenyltetrazolium bromide] assay. The OD values were read using the BioTek Synergy H4 Plate Reader, BioTek, USA. Results are expressed as a percentage of viability relative to control cells (incubated in control extract).

### 2.4 Evaluation of Mutagenicity and Genotoxicity *In Vitro*

The mutagenicity and genotoxicity of the Curdlan-based biomaterial were evaluated in accordance with the ISO 10993-3:2014 standard,^34^ while the liquid extracts were prepared according to the ISO 10093-12:2012 standard.^37^ Thus, the bacterial reverse mutation test (based on the OECD 471 guideline), *in vitro* mammalian chromosomal aberration test (based on OECD 473 guideline), and *in vitro* mammalian cell micronucleus test (based on OECD 487 guideline) were performed. Additionally, a comet assay was conducted.

#### 2.4.1 Bacterial Reverse Mutation Test (Ames Assay)

The Ames test (*Salmonella typhimurium* reverse mutation assay) was performed by a certified laboratory of the National Medicines Institute (Warsaw, Poland). For this purpose, 5 bacterial strains of *S. typhimurium*, namely, TA97 (CIP 108115), TA98 (CIP 103798), TA100 (CIP 103799), TA102 (CIP 104406), and TA1535 (CIP 103793), were used. The biomaterial extract was prepared in phosphate-buffered saline (PBS) in the proportion of 100 mg/1 mL (72 h, 37 °C). Bacterial strains were cultured with a biomaterial extract and a control extract (PBS incubated without the biomaterial) in the presence and absence of a metabolic activator, namely, cofactor-supplemented postmitochondrial factor (S9). In addition, positive controls were included. *S. Typhimurium* incubated without metabolic activation (-S9) were stimulated with 4-nitro-1,2-phenylendiamine (TA97, TA98, TA100, and TA1535 strains) or methylmethanesulfonate (TA102 strain). In turn, bacteria incubated with metabolic activation (+S9) were stimulated with 2-aminoanthracene (TA97, TA98, TA100, and TA1535 strains) or 9-aminoacridine (TA109 strain). Results were presented as a mean number of revertants obtained in tests without metabolic activation (−S9) and with metabolic activation (+S9). The number of revertants obtained in the experiment was compared with the number of spontaneous revertants (the fold change is given in brackets).

#### 2.4.2 *In Vitro* Mammalian Chromosomal Aberration Test

This experiment was performed by using the CHO-K1 cell line. Briefly, the cells were seeded into 6-well plates in a culture medium at a concentration of 1 × 10^5^ cells/well. The liquid extract from the biomaterial was prepared in the same way as for cytotoxicity evaluation (Section 2.3.). The cells were treated with the control extract as well as the biomaterial extract for long and short incubation times without (−S9) and with (+S9) metabolic activation, respectively. Thus, during the long-term evaluation, cells were treated for 24 h with a control extract without S9 (−S9), a biomaterial extract without S9 (−S9), and a mitomycin C solution at a concentration of 0.25 μg/mL without S9 (−S9). In turn, during a short exposure time (5 h), cells were incubated with the control extract without S9 (−S9), biomaterial extracts with and without S9 (+S9 and −S9, respectively), mitomycin C solution at concentrations of 0.5 μg/mL without S9 (−S9, first positive control), and 3 μg/mL cyclophosphamide solution with S9 (+S9, second positive control). Then, the CHO-K1 cells were incubated for 3 h with a solution of colcemide at a concentration of 0.1 μg/mL in order to arrest cells in metaphase. Next, the cells were detached and swollen by incubation with potassium chloride solution at a concentration of 0.075 M and then fixed with a solution of methanol and acetic acid in a ratio of 3:1. Next, the cells were stained with 0.7% Giemsa solution and analyzed using an upright microscope (Eclipse Ni-U, Nikon Instruments, USA). 100 cells per sample were analyzed.

#### 2.4.3 *In Vitro* Mammalian Cell Micronucleus Test

This experiment was carried out under the same conditions as described in Section 2.4.2. After incubation, the CHO-K1 cells were treated for 24 h with a 3 μg/mL cytochalasin B solution in order to identify binucleated (BN) cells. After this time, the cells were stained with a 50 μg/mL acridine orange solution and immediately observed under a confocal laser scanning microscope (CLSM, Olympus Fluoview equipped with FV1000, Olympus, Japan). The induction of micronuclei (MN) was determined in at least 1000 BN cells. The BNMN was scored by calculating the BN micronucleated cell frequency as the number of BN cells containing one or more MN per 1000 BN cells. The cytokinesis-block proliferation index (CBPI) was calculated according to the OECD 487 guideline using the following formula
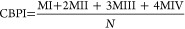
where MI, MII, MIII, and MIV denotes the number of mono-, bi-, tri-, and multinucleated cells, respectively. In turn, *N* denotes the number of scored cells.

#### 2.4.4 Comet Assay

This experiment was carried out under the same conditions as described in Section 2.4.2. The comet assay was carried out using a commercially available assay (Oxi Select 96-Well Comet Assay Slides) in accordance with the manufacturer’s guidelines. The cells were analyzed under an inverted fluorescence phase contrast microscope (Olympus CKX53, Olympus, Japan) using an FITC filter (100 cells per sample were evaluated).

### 2.5 Evaluation of Viability and Proliferation of Human Chondrocytes and Human Osteoblasts *In Vitro*

The viability and proliferation of human chondrocytes (hChon cells) and human fetal osteoblasts (hFOB 1.19 cell line) were evaluated in direct contact with biomaterials (biomaterial discs were obtained from the top layer of the biomaterial and the lower layer of the biomaterial). The hChon cells were isolated from articular cartilage with the consent of the Bioethics Committee of the Medical University of Lublin, Poland, no. KE-0254/114/2020 from June 2020, according to the procedure described in detail by us earlier.^38^ The hChon cells were cultured in the DMEM/F12 1:1 medium with the addition of 10% FBS, 10 ng/mL hFGF-2, 1 ng/mL hTGF-β1, 10 U/mL penicillin, and 10 μg/mL streptomycin at 37 °C (Heraeus cytoperm 2 CO_2_ 6-Door Incubator, Thermo Fisher Scientific, USA), while hFOB 1.19 cells were maintained in accordance with ATCC guidelines, namely, in the DMEM/Ham’s F12 medium enriched with 300 μg/mL G418, 10% FBS, 100 U/mL penicillin, and 100 μg/mL streptomycin at 34 °C (ESCO CelCulture CO_2_ Incubator, ESCO Lifesciences Group, Asia). To assess cell viability, hChon and hFOB 1.19 cells were seeded on biomaterials at concentrations of 4 × 10^5^ cells/sample and 2 × 10^5^ cells/sample, respectively. After 48 and 72 h of incubation, cells were stained using the Live/Dead Cell Double Staining Kit and visualized using a CLSM (Olympus Fluoview equipped with FV1000, Olympus, Japan). Subsequently, to evaluate cell proliferation, hChon and hFOB 1.19 cells were seeded on biomaterials at a concentration of 2 × 10^5^ cells/sample and 1 × 10^5^ cells/sample, respectively. After 4 and 7 days of incubation, the metabolic activity of the cells was estimated using the WST-8 assay. Moreover, to assess cell morphology, after 7 days of incubation, cell nuclei and actin filaments of the cytoskeleton were stained using Hoechst 33342 fluorescent dye and AlexaFluor 635 Phalloidin, respectively. The cells were observed under a CLSM (Olympus Fluoview equipped with an FV1000, Olympus, Japan). For the assessment of both viability and proliferation, cells grown on polystyrene (PS) served as the experimental control.

### 2.6 Evaluation of Inflammatory Response *In Vitro*

In order to assess the inflammatory response to biomaterials *in vitro*, human acute monocytic leukemia cells (THP-1 cell line) were used. The experiments were conducted according to ref (39) with some modifications. Briefly, cells were cultured in RPMI-1640 medium with the addition of 10% FBS, 0.05 mM mercaptoethanol, 100 U/mL penicillin, and 100 μg/mL streptomycin at 37 °C. Then, THP-1 cells at a concentration of 2 × 10^5^ were seeded on biomaterials in a complete culture medium enriched with 200 nM phorbol 12-myristate 13-acetate (PMA) and cultured for 48 h to stimulate the process of differentiation into macrophages. Next, the differential medium was replaced with a complete culture medium, and cells were incubated for 2, 4, and 6 days. Cells grown on PS were served as negative control (Control-), while cells cultured on PS in the presence of 100 ng/mL LPS and 20 ng/mL interferon gamma were considered as a positive control (Control+). The concentration of IL-6, MIP-1β, and TNF-α produced by THP-1-derived macrophages was evaluated by ELISA tests using the BioTek Synergy H4 Plate Reader, BioTek, USA.

### 2.7 *Ex Vivo* Model of Human Osteochondral—OC—Defect

*Ex vivo* studies were carried out using human osteoarthritic cartilage biopsies (12 mm diameter, Mankin score 7–9) collected from patients undergoing total knee arthroplasty (*n* = 7, ages 72–78) with informed consent and according to the Helsinki Declaration. The study was approved by the Ethics Committee of the Saarland Physicians Council (No. 270-17). The following research groups were included in this study: control biopsies, biopsies where an experimental osteochondral defect was created with a 8 mm punch (Kai Europe, Solingen, Germany) and left empty,^40^ and biopsies where an experimental osteochondral defect was created with a 8 mm punch and filled with the Curdlan-based biomaterial. The obtained biopsies were incubated in the culture medium (Dulbecco’s modified Eagle’s medium supplemented with 10% FBS, 100 U/mL penicillin G, and 100 μL/mL streptomycin) at 37 °C (5% CO_2_, 95% humidity) for 1, 3, 7, 10, and 14 days. Subsequently, the concentration of IL-6, MIP-1β, and TNF-α in the supernatants from the biopsies was evaluated using specific ELISA tests and a GENios spectrophotometer/fluorometer (Tecan, Crailsheim, Germany). On day 14, the biopsies were collected for the histological analyses. Briefly, the samples were fixed in formaldehyde (4%), washed in PBS, decalcified using 10% sodium citrate/25% formic acid for 4 weeks at room temperature, and dehydrated in graded alcohol.^40^ Histological analyses were performed on paraffin-embedded sections of the biopsies (10 μm) by safranin O staining (matrix proteoglycans).^40^ The samples were analyzed under light microscopy (Olympus BX 45, Olympus, Germany) to estimate the tissue integration of the biomaterial.

### 2.8 Evaluation of Toxicity toward Zebrafish (*D. rerio*)

*In vivo* studies using zebrafish larvae and adults (line AB) were performed with the approval of the Local Ethics Committee for Animal Experimentation in Lublin, Poland, with consent no. 102/2022. The experiments were carried out using liquid extracts obtained from the biomaterials. For this purpose, biomaterials were immersed in the E3 medium (studies with the use of zebrafish larvae) or system water (studies with the use of zebrafish adults) according to the same procedure as described in Section 2.3. Liquids incubated under the same conditions without biomaterials served as experimental controls. The experiments were carried out in the Experimental Medicine Centre, Medical University of Lublin (Poland), in accordance with the National Institute of Health Guidelines for the Care and Use of Laboratory Animals and the European Community Council Directive for Care and Use of Laboratory Animals of 22 September 2010 (2010/63/EU).

#### 2.8.1 Assessment of Toxicity in Zebrafish Larvae

This experiment was performed using zebrafish larvae at 7 days post fertilization (7 dpf). Briefly, immediately after fertilization (0 dpf), zebrafish embryos (10 per group) were transferred to 96-well plates and incubated with control extracts and biomaterial extracts at 28.5 °C (Incubator IN110—Memmert GmbH + Co. Deutschland). On the third day of the experiment, the extracts were replaced with the fresh ones. After 7 days of incubation, the larvae were euthanized with 0.3–0.5 mg/mL tricaine solution, fixed with 4% paraformaldehyde overnight, and stained overnight with 0.02% Alcian blue solution in 70% ethanol containing 200 mM magnesium chloride. Then, the larvae were bleached with 3% hydrogen peroxide with 1% potassium hydroxide for 30 min and cleared using 80% glycerol with 0.1% potassium hydroxide. Stained larvae were visualized using a Zeiss Axio Vert stereomicroscope (Zeiss, Germany). The effect of the extracts on cartilage development in zebrafish larvae was assessed by measurements of the following parameters: distance between anterior Meckel’s cartilage and anterior ceratohyal (a), left anterior Meckel’s posterior palatoquadrate length (b), right anterior Meckel’s posterior palatoquadrate length (c), distance between anterior palatoquadrates (d), and ceratohyal angle (e). Analysis was carried out using ImageJ 1.52v software and CorelDRAW X7 software version 17.6.0.1021.

#### 2.8.2 Assessment of Regeneration of Amputated Caudal Fin in an Adult Zebrafish

This experiment was conducted using adult 3 month-old zebrafish (10 per group). Biomaterial extracts and control extracts were prepared as described in Section 2.7.1. but using system water. The zebrafish’s caudal fins were clipped using a surgical scalpel. Two fish were maintained in one tank split into two equal parts with a divider in the presence of 500 mL of control extract or biomaterial extract. Extracts were replaced with the new ones every 3 days. After 7, 14, and 21 days, the zebrafish were anesthetized in 168 g/L tricaine solution, and their caudal fins were visualized using a Zeiss Axio Vert stereomicroscope (Zeiss, Germany). Measurements of caudal fin growth over time were made using ImageJ 1.52v software and CorelDRAW X7 software, version 17.6.0.1021.

### 2.9 Application of the Biomaterial in Veterinary Patients with OCD

Three dogs took part in the experimental therapy using the Curdlan-based biomaterial (examples of intraoperative photos during the implantation procedure are shown in Figure S1). The dog owners gave written consent to the implantation of biomaterials. The first case concerned a 10 month-old St. Bernard named Tadzik. The dog was brought to the veterinary clinic with symptoms of lameness. Therapy with nonsteroidal anti-inflammatory drugs (NSAIDs) did not bring any improvements. The patient did not allow himself to be examined without sedation. On clinical examination after sedation, there is marked tenderness in both hip joints. X-ray examination showed OCD of the right shoulder joint (Figure S2A). Tadzik underwent an operation involving the removal of damaged cartilage along with a fragment of subchondral bone and then implantation of the Curdlan-based biomaterial. X-rays were taken immediately after the procedure and after 1 and 3 months (Figure S2B–D, respectively). The second case concerned a 1 year-old Border Collie named Ellie. On orthopedic examination, there was pain in the right shoulder joint and slightly in the right elbow in hyperextension. The female dog also suffered from lameness in the right shoulder joint resulting from OCD, as proven by X-ray examination (Figure S3A). In this case, therapy with NSAIDs also did not bring any results. Therefore, a procedure was performed to remove the damaged cartilage along with a fragment of the subchondral bone, and then a Curdlan-based biomaterial was implanted. X-rays were taken immediately after the procedure and after 1 and 3 months (Figure S3B–D). The third case involved a 6 month-old Bernese mountain dog named Bruno. The right front paw was severely painful during forced movements in the shoulder joint. Similarly to earlier cases, Bruno had lameness due to OCD of the right shoulder joint (Figure S4A). After ineffective therapy with NSAIDs, a decision was made to remove damaged cartilage together with a fragment of the subchondral bone and to implant a Curdlan-based biomaterial. X-rays were taken immediately after the procedure and after 1 and 3 months as well as after 12 months (Figure S4 B–E).

### 2.10 Statistical Analysis

Each experiment was performed using at least 3 independent biomaterial samples. The obtained data were demonstrated as mean values ± the standard deviation. The normal distribution of results was evaluated by a D’Agostino and Pearson omnibus normality test. Then, an unpaired Student’s *t*-test or a one-way ANOVA test, followed by a Tukey’s multiple comparison test or a two-way ANOVA test, followed by a Bonferroni comparison test were carried out (GraphPad Prism 5, Version 5.04 Software). Differences were considered statistically significant when the *P* value was less than 0.05 (*P* < 0.05).

## 3 Results and Discussion

### 3.1 Cytotoxicity *In Vitro*

**Figure 1:**
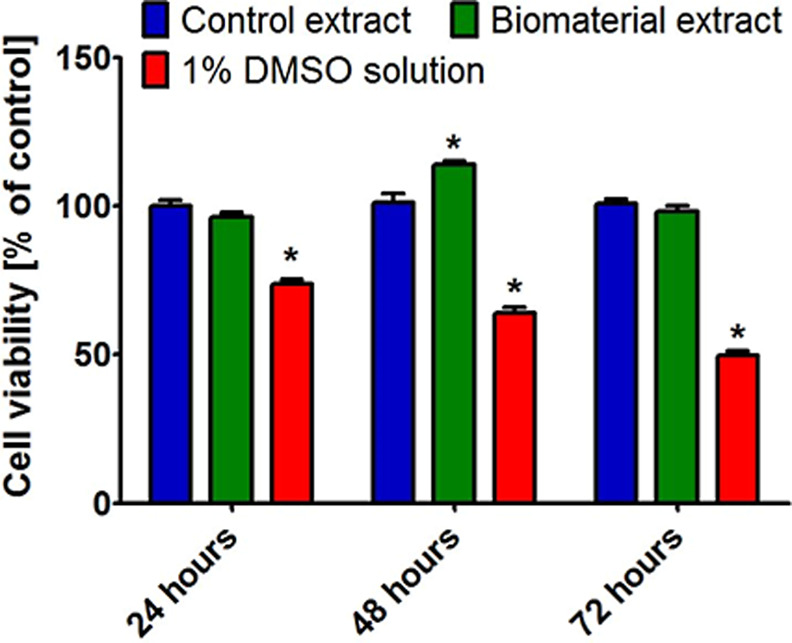
Evaluation of cytotoxicity of a Curdlan-based osteochondral biomaterial. The experiment was carried out using liquid extracts obtained from the biomaterial and PS (control extract). A 1% DMSO solution was used as a positive control of cytotoxicity. After 24, 48, and 72 h of incubation, CHO-K1 cell viability was assessed using MTT assay. * Statistically significant differences compared to control extract (a two-way ANOVA test, followed by a Bonferroni comparison test, *P* < 0.05).

The *in vitro* cytotoxicity assessment according to the ISO 10993-5:2009 standard is the first mandatory test for potential medical devices.^33^ According to these guidelines, a biomaterial extract can be considered noncytotoxic if it does not decrease cell viability below 70% compared with the control. After 24, 48, and 72 h of incubation, the MTT test showed that the extracts obtained from the Curdlan-based biomaterial did not reduce the viability of CHO-K1 cells (Figure 1). Thus, cell viability after incubation with biomaterial extracts was close to 100% or even slightly higher (*P* < 0.05 after 48 h of incubation) compared to the viability of cells incubated with control extracts. In turn, the viability of cells incubated with 1% DMSO solution (positive control of cytotoxicity) was significantly reduced (*P* < 0.05) compared to the viability of cells incubated with both the control extract and the biomaterial extract. Therefore, taking into account the obtained results, the Curdlan-based biomaterial should be considered as noncytotoxic *in vitro*. It is worth emphasizing that the cytotoxicity test of biomaterials using liquid extracts (i.e., evaluation of cell viability in indirect contact with biomaterials) according to the ISO 10993-5:2009 standard is very often carried out by researchers.^41−44^ Importantly, the ISO 10993-5:2009 standard indicates that extracts should be prepared according to the ISO 10093-12:2012 standard. The method of obtaining the extract depends largely on the type of the biomaterial, its properties, and application or storage method. It is indicated that the extract from the biomaterial can be obtained under the following conditions: 1) 24 ± 2 h, 37 ± 1 °C; 2) 72 ± 2 h, 50 ± 2 °C; 3) 24 ± 2 h, 70 ± 2 °C; and 4) 1 ± 0.2 h, 121 ± 2 °C. In our study, we prepared the extract from the Curdlan-based biomaterial based on the first conditions because they are most often used by researchers, which results from the fact that an implantable biomaterial will be located in the living body, where the temperature is around 37 °C.^45^ In addition, depending on the type of the biomaterial, the extract should be prepared in the appropriate proportion, namely, taking into account the surface area of the biomaterial or its mass. For a porous biomaterial with shape similar to a cylinder (such as osteochondral scaffolds), which very often can release substances into the surrounding environment, it is justified to assume a proportion of 0.1 g/1 mL of culture medium, as we did in our study. What is more, the ISO standard suggests choosing cell lines obtained from well-known and proven repositories, such as ATCC. In justified cases, the use of primary cells is also permissible. In turn, for the assessment of the cytotoxicity of the extract, the ISO 10993-5:2009 standard recommends the use of the four most popular assays for assessing cell viability, i.e., the neutral red uptake cytotoxicity test, colony formation cytotoxicity test, MTT cytotoxicity assay, or XTT cytotoxicity test. In our study, we used the MTT test as suggested by the ISO 10993-5:2009 standard, but other less popular tests for assessing cell viability (e.g., resazurin-based test or lactate dehydrogenase cytotoxicity test) can also be used.^42^ Based on the available scientific literature, it can be seen that cytotoxicity studies of osteochondral biomaterials according to the ISO 10993-5:2009 standard using liquid extracts are conducted under different conditions. For example, Aydin et al.^46^ evaluated the cytotoxicity of an extract from a three-layer osteochondral biomaterial consisting of poly(l-lactide) (PLLA), poly(ε-caprolactone) (PCL), collagen type I, and hydroxyapatite. The authors prepared an extract from the biomaterial (72 h, 37 °C) and evaluated its cytotoxicity after 24 h of incubation against mouse fibroblasts (L929 cell line) using the MTT test. The obtained results showed that the extract did not show cytotoxicity *in vitro* because the cell viability was about 100% compared to the control. Lee et al.^47^ prepared an extract from a 3D-printed bioceramic osteochondral scaffold (72 h, 37 °C) and evaluated its cytotoxicity toward mouse fibroblasts (NIH-3T3 cell line) using also MTT assay. After 24 and 48 h of incubation with the extract, the cell viability was about 95% and 94% compared to control cells. Fang et al.^48^ assessed the cytotoxicity of an extract from the extracellular matrix (ECM)-based biphasic osteochondral scaffold enriched with kartogenin and metformin. The extract was obtained by incubation for 24 h at 37 °C and tested against mouse fibroblasts (L929 cell line). After 24 h of incubation with the extract, the cell viability was close to 100% compared to the control (based on WST-1 assay). In turn, Asensio et al.^49^ estimated the cytotoxicity of extracts obtained from the osteochondral scaffold containing hyaluronic acid and Sr/Zn folates. Extracts were collected after 1, 2, 7, and 14 days of incubation at 37 °C. The viability of human osteoblast (hOB cell line) and human chondrocytes (hAC cell line) treated with extracts was assessed after 24 h of incubation using the AlamarBlue test (resazurin-based assay). The obtained results showed that the extracts not only did not show cytotoxicity but some of them even stimulated cell viability compared to the control. These examples therefore demonstrate that cytotoxicity testing in an indirect contact, using liquid extracts, can be used for the preliminary assessment of cytocompatibility of biomaterials, including osteochondral scaffolds. It is worth emphasizing, however, that it would be beneficial if improved versions of ISO standards provided guidelines for biomaterials with specific applications, e.g., implantable materials such as cartilage or bone scaffolds. This would allow for greater comparability of the results between scientists.

### 3.2 Mutagenicity and Genotoxicity *In Vitro*

In addition to assessing cytotoxicity, mutagenicity and genotoxicity *in vitro* of scaffolds should be evaluated, especially if they are designed for long-term use. The biomaterials are considered mutagenic if they have the ability to cause mutations. Moreover, scaffolds are characterized by genotoxicity when they cause deoxyribonucleic acid (DNA) damage and other disorders that may indirectly affect the functioning of genes. Both mutagenic and genotoxic biomaterials should not be used clinically.^50^ In this study, mutagenicity *in vitro* was assessed by the Bacterial Reverse Mutation Test (Ames assay), while genotoxicity was estimated using the *in vitro* chromosomal aberration test, the *in vitro* micronucleus (MNvit) assay, and the *In Vitro* Comet Test.

The Ames test (Table 1) showed that the number of revertants after incubation with the Curdlan-based biomaterial extract was not significantly higher (*P* < 0.05) compared to the number of spontaneous revertants, both without (in the absence of S9) and with (in the presence of S9) metabolic activation. In turn, the number of revertants after incubation with positive reference substances was significantly higher (*P* < 0.05) compared to the number of spontaneous revertants. Unambiguous results were obtained toward five tested bacterial strains. According to ISO 10993-3:2014 standard guidelines,^34^ a sample is considered nonmutagenic if it does not cause at least a 2-fold increase in the level of reversion compared to the level of spontaneous reversion in none of the bacterial strains. Thus, considering the lack of a significant increase in the level of reversion (multiplicity is shown in Table 1) after incubation with Curdlan-based biomaterial extracts compared to the level of spontaneous reversion, this biomaterial should be considered nonmutagenic.

**Table 1:** Results of Bacterial Reverse Mutation Test (Ames Assay) Expressed as the Number of Revertants after Incubation with the Curdlan-Based Biomaterial Extract^a^

Salmonella typhimurium strain	test without metabolic activation (−S9)			test with metabolic activation (+S9)		
	spontaneous reversion	positive control	biomaterial extract	spontaneous reversion	positive control	biomaterial extract
TA97	164 ± 14	1315 ± 273(8.01×)*	222 ± 23(1.35 ×)	176 ± 5	1241 ± 37(7.05×)*	206 ± 14(1.17×)
TA98	35 ± 10	1807 ± 180(51.62×)*	31 ± 7(0.90×)	39 ± 8	1091 ± 88(27.74×)*	36 ± 3(0.91×)
TA100	118 ± 4	1114 ± 87(9.46×)*	123 ± 16(1.04×)	121 ± 14	1547 ± 304(12.82×)*	118 ± 17(0.97×)
TA102	305 ± 9	2639 ± 105(9×)*	289 ± 28(0.95×)	313 ± 6	2536 ± 30(8×)*	338 ± 19(1.08×)
TA1535	19 ± 10	89 ± 13(4.59×)*	17 ± 2(0.85×)	18 ± 6	311 ± 14(17.28×)*	17 ± 7(0.95×)

a The experiment was performed without (−S9) and with (+S9) metabolic activation. Fold increase in the level of reversion was presented in brackets. * Statistically significant differences compared to the number of spontaneous revertants, unpaired Student’s *t*-test (*P* < 0.05).

The *in vitro* chromosomal aberration test indicated that Curdlan-based biomaterial extracts did not exhibit any change in chromosomal aberration (Figure S5A) compared to the control extract. A marked increase in aberration in the chromatid region, predominantly chromatid breaks (Figure S5B), fragments, quadriradial (Figure S5C), and deletion (Figure S5D) was observed in the positive controls, i.e., cells treated with mitomycin C at a concentrations of 0.25 μg/mL (long-term incubation) and 0.5 μg/mL (short-time incubation) as well as with cyclophosphamide at a concentration of 3 μg/mL (short-time incubation), indicating validation of the test system and experimental procedure. Moreover, numerical aberration, especially loss of chromosomes, was observed (Figure S5E). Table 2 summarizes the number of all identified chromatid- and chromosome-type aberrations. Thus, this experiment revealed that Curdlan-based biomaterial extracts did not possess the ability to induce chromosomal aberrations in mammalian cells *in vitro*.

**Table 2:** Results of *In Vitro* Mammalian Chromosomal Aberration Test Expressed as the Number of Structural Aberrations (Chromatid Type and Chromosome Type)^a^

structural aberrations					
Chromatid					
	chromatid gap	chromatid break	fragment	deletion	exchange
Long-term treatment					
control extract	0	0	0	0	0
biomaterial extract	0	0	0	0	0
mitomycin C 0.25 g/mL	3	8	6	10	2
Short-term treatment					
control extract	0	0	0	0	0
biomaterial extract (−S9)	0	0	0	0	0
biomaterial extract (+S9)	0	0	0	0	0
mitomycin C 0.5 μg/mL (−S9)	3	6	4	11	3
cyclophosphamide 3 μg/mL (+S9)	4	5	4	10	4
Chromosome					
	isochromatid gap	chromosome break	acentric fragment	deletion	exchange
Long-term treatment					
control extract	0	0	0	0	0
biomaterial extract	0	0	0	0	0
mitomycin C 0.25 μg/mL	0	5	0	4	8
Short-term treatment					
control extract	0	0	0	0	0
biomaterial extract (−S9)	0	0	0	0	0
biomaterial extract (+S9)	0	0	0	0	0
mitomycin C 0.5 μg/mL (−S9)	0	6	0	5	10
cyclophosphamide 3 μg/mL (+S9)	0	3	0	7	9
numerical aberrations					
	pulverized chromosomes and pulverized cell	cell with greater than 10 aberrations	endoreduplication	polyploidy	aneuploidy
Long-term treatment					
control extract	0	0	0	0	0
biomaterial extract	0	0	0	0	0
mitomycin C 0.25 μg/mL	0	0	0	0	7
Short-term treatment					
control extract	0	0	0	0	0
biomaterial extract (-S9)	0	0	0	0	0
biomaterial extract (+S9)	0	0	0	0	0
mitomycin C 0.5 μg/mL (-S9)	0	0	0	0	8
cyclophosphamide 3 μg/mL (+S9)	0	0	0	0	15

a The experiment was performed without (−S9) and with (+S9) metabolic activation. Structural changes were observed after staining the CHO-K1 cells with 0.7% Giemsa solution.

The MNvit assay demonstrated no difference in CHO-K1 cells treated with Curdlan-based biomaterial extracts (both after long-term and short-term treatment) compared to cells incubated with the control extract (Table 3). Thus, the MN values, BNMN, and CBPI indexes of CHO-K1 cells exposed to biomaterial extracts were similar to those obtained for control cells. For both groups (Figure S6 A,B), dividing cells in anaphase (Figure S6C), telophase (Figure S6D), and metaphase (Figure S6C) were visualized. In turn, an increase in all investigated parameters was observed for cells incubated with positive controls, namely, mitomycin C at a concentration of 0.25 and 0.5 μg/mL and cyclophosphamide at a concentration of 3 μg/mL (Table 3). Thus, microscope observation showed MN formation (Figure S6 E) and displayed characteristic apoptosis in cells (Figure S6 F) incubated with positive reference substances. Moreover, nuclear convulsion, fragmentation, cytoplasmic blebbing, giant cells, and cytoplasmic vacuolation were observed (Figure S6 G,H). Therefore, this test revealed that Curdlan-based biomaterial extracts did not have chromosome-damaging potential *in vitro*.

**Table 3:** Results of *In Vitro* Mammalian Cell Micronucleus Test Expressed as the Frequency of MN, Micronucleated BN Cells, and Cytokinesis-Block Proliferation Index in CHO-K1 Cells^a^

	total MN	BNMN ‰	CBPI
Long-term treatment			
control extract	1	1	1.84
biomaterial extract	1	0	1.85
mitomycin C 0.25 μg/mL	17	15	1.97
Short-term treatment			
control extract	1	1	1.84
biomaterial extract (−S9)	1	2	1.84
biomaterial extract (+S9)	2	2	1.85
mitomycin C 0.5 μg/mL (-S9)	19	23	1.96
cyclophosphamide 3 μg/mL (+S9)	29	33	1.99

a The experiment was performed without (−S9) and with (+S9) metabolic activation. Changes were observed after staining the CHO-K1 cells with acridine orange solution. MN—number of MN in 1000 BN cells; BNMN ‰—per mille of BN cells with MN in 1000 BN cells; and CBPI—cytokinesis-block proliferation index.

The *In Vitro* Comet Test showed no differences in comet formation—DNA tail presence—both in CHO-K1 cells treated for long and short periods with control extract and Curdlan-based biomaterial extract (Table 4). The increase of DNA damage was observed in cells incubated with all positive reference substances (mitomycin C 0.25 and 0.5 μg/mL as well as cyclophosphamide 3 μg/mL). Representative images are shown in Figure S7A–E. This assay proved that the Curdlan-based extract did not cause DNA strand breaks in mammalian cells *in vitro*.

**Table 4:** Results of *In Vitro* Comet Test Expressed as a Percentage of DNA Tail Presence in CHO-K1 Cells^a^

	presence of tail DNA [%]
Long-term treatment	
control extract	3
biomaterial extract	2
mitomycin C 0.25 μg/mL	30
Short-term treatment	
control extract	2
biomaterial extract (−S9)	3
biomaterial extract (+S9)	3
mitomycin C 0.5 μg/mL (−S9)	29
cyclophosphamide 3 μg/mL (+S9)	35

a The experiment was performed without (−S9) and with (+S9) metabolic activation. The test was conducted by using a commercially available kit (Oxi Select 96-Well Comet Assay Slides).

Based on the available literature, it is observed that the genotoxic and mutagenic effect of extracts from biomaterials according to the ISO 10993-3:2014 standard is less frequently assessed than cytotoxicity according to the ISO 10993-5:2009 standard. This phenomenon may result from a more complicated methodology and a greater degree of difficulty in interpreting the obtained results. Similar to the ISO 10993-5:2009 standard, the ISO 10993-3:2014 standard indicates the preparation of liquid extracts from biomaterials based on the ISO 10093-12:2012 standard (variables in the preparation of extracts are described in Section 3.1.) and then the assessment of mutagenicity using the bacterial reverse Mutation Test (Ames assay) and genotoxicity using the *in vitro* chromosomal aberration test and the MNvit assay. In addition to cytotoxicity evaluation, muta- and genotoxicity studies are very crucial for assessing the cytocompatibility of the biomaterial because substances released from the biomaterial may not reduce cell viability but cause mutations or DNA damages.^51^ Although, as mentioned above, there are studies where mutagenicity and genotoxicity of biomaterials were studied,^45,51−54^ no reports were found where these analyses were performed to assess specifically osteochondral scaffolds. We therefore believe that our work will set new trends and convince scientists to assess the mutagenicity and genotoxicity of new osteochondral scaffolds as they complement cytotoxicity studies.

### 3.3 Viability and Proliferation of Human Chondrocytes and Human Osteoblasts *In Vitro*

**Figure 2:**
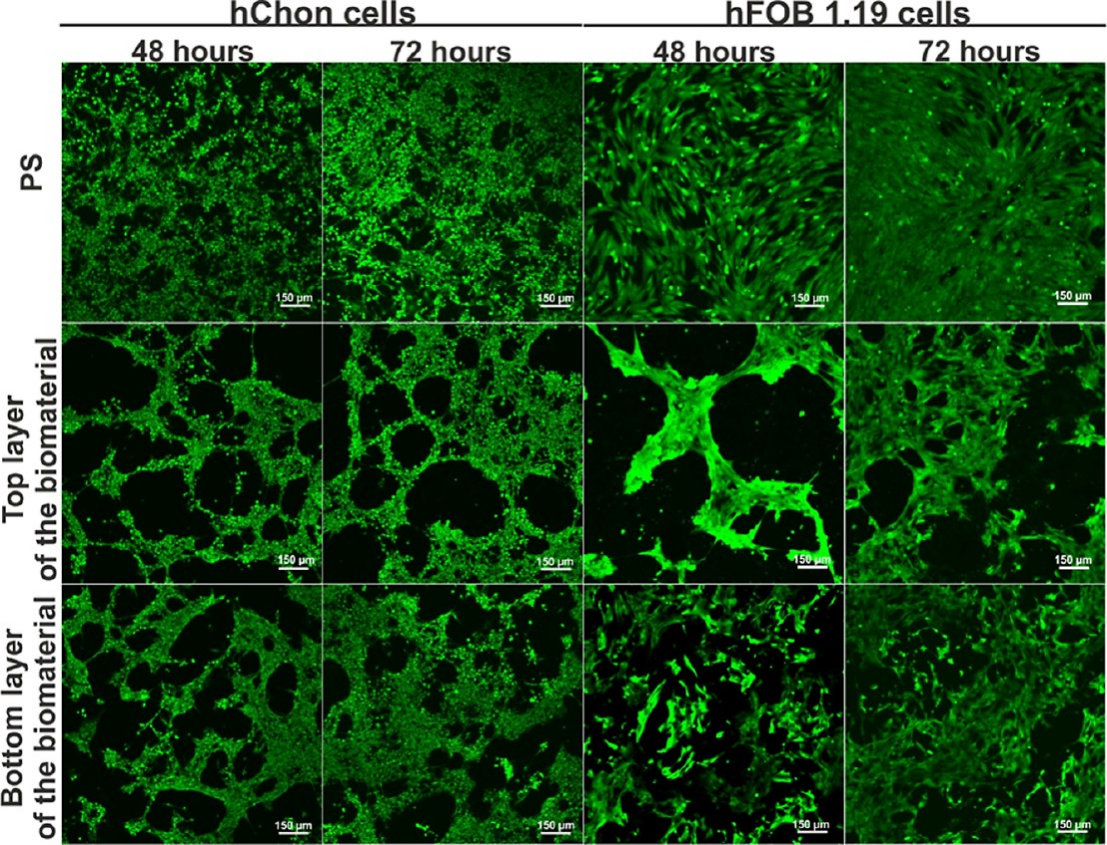
Viability of human chondrocytes (hChon cells) and human osteoblasts (hFOB 1.19 cells) cultured on PS (experimental control), on the top layer of the Curdlan-based biomaterial, and on the bottom layer of the Curdlan-based biomaterial. Live cells—green fluorescence; dead cells—red fluorescence. Magnification: 100×, scale bar: 150 μm.

**Figure 3:**
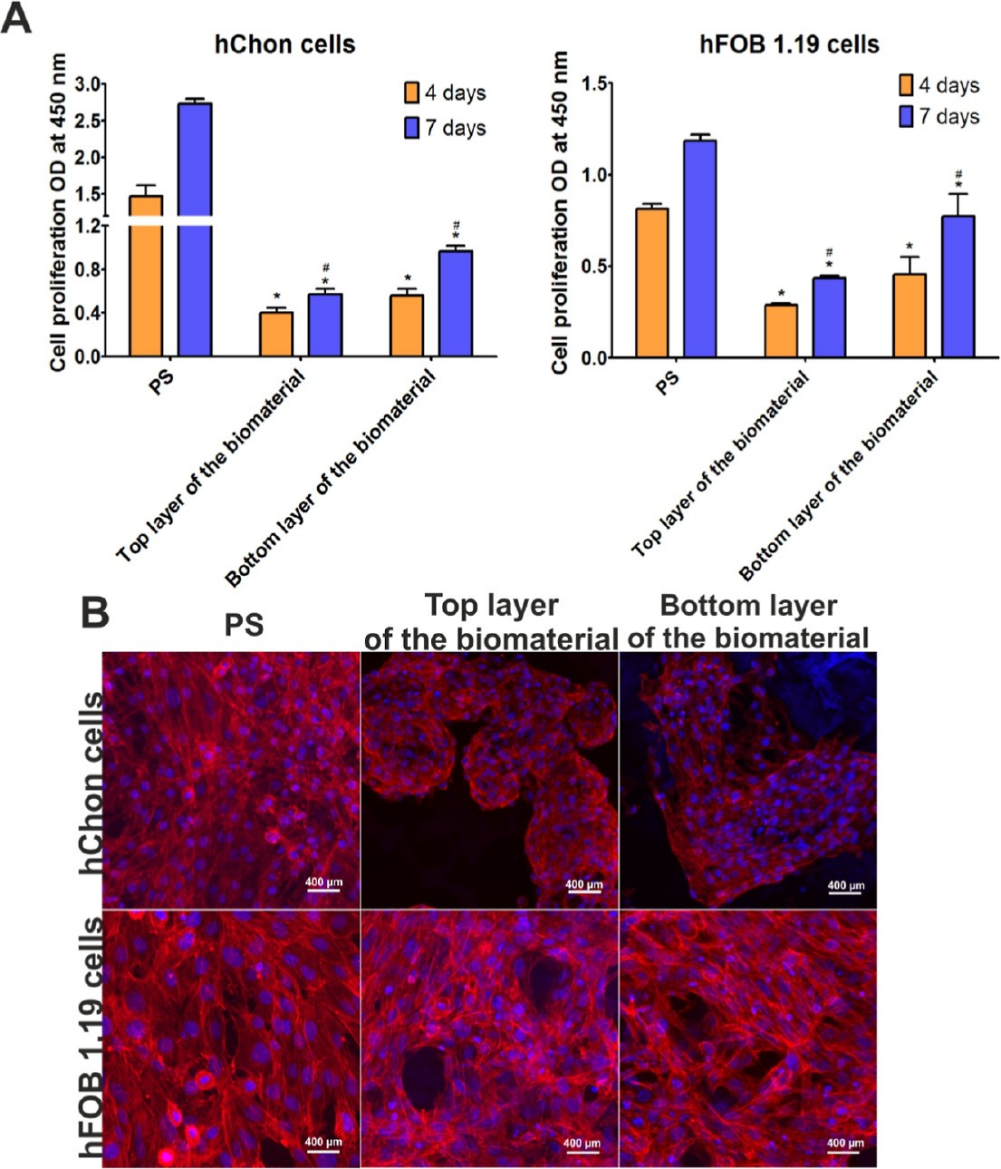
Proliferation of human chondrocytes (hChon cells) and human osteoblasts (hFOB 1.19 cells) cultured on PS (experimental control), on the top layer of the Curdlan-based biomaterial, and on the bottom layer of the Curdlan-based biomaterial. Results obtained with the WST-8 test (A). * Statistically significant differences compared to cells cultured on PS. ^#^ Statistically significant differences compared to cells cultured on the top layer of the Curdlan-based biomaterial (a two-way ANOVA test, followed by a Bonferroni comparison test, *P* < 0.05). Images obtained under the CLSM (B); cell nuclei—blue fluorescence; and F-actin filaments of cytoskeleton—red fluorescence. Magnification: 200×, scale bar: 400 μm.

Chondrocytes are the most numerous cell type in cartilage, and their role is entirely focused on the turnover of ECM of this tissue.^55^ Although cartilage possesses poor ability to self-repair, after injury is able to regenerate thanks to chondrocyte proliferation and ECM synthesis.^56^ In turn, the subchondral bone includes several types of cells, such as osteoblasts, osteoclasts, osteocytes, and stem cells.^55^ Osteoblasts are bone-forming cells and play a huge role in bone remodeling, *inter alia* via production of many bone matrix proteins.^57^ Bioactive OC scaffolds should therefore simultaneously support the colonization and proliferation of chondrocytes and osteoblasts.^58,59^ For this reason, in this study, the response of these cells in direct contact with the Curdlan-based scaffold was assessed. After 48 and 72 h of incubation, microscopic observations showed that both hChon and hFOB 1.19 cells colonized the surface of the biomaterials (Figure 2). Both cell types were viable (emitted green fluorescence) and grew on both the top and bottom biomaterial layers, indicating that the whole biomaterial promotes the growth of human chondrocytes and human osteoblasts. Moreover, the WST-8 test showed that both the top layer of the biomaterial and the bottom layer of the biomaterial promoted the division of hChon and hFOB 1.19 cells because their metabolic activity increased with the length of the experiment (Figure 3A). After 7 days of incubation, the CLSM observations (Figure 3B) demonstrated that both cell types cultured on the top layer of the biomaterial and on the bottom layer of the biomaterial had a well-developed cytoskeleton, which confirms that the whole surface of the Curdlan-based biomaterial supports the colonization and growth of human chondrocytes and human osteoblasts *in vitro*. In our previous study,^32^ we indicated that the Curdlan-based biomaterial promoted the adhesion, proliferation, and chondrogenic as well as osteogenic differentiation of human mesenchymal stem cells. Thus, the results obtained in this study are in good agreement with the data obtained earlier and confirm the high *in vitro* cytocompatibility of the Curdlan-based biomaterial.

In addition to assessing the cytotoxicity of biomaterials in indirect contact (using liquid extracts), it is crucial to also assess the response of cells in direct contact with the biomaterials (after the cells are seeded on the biomaterial). The evaluation of the cytotoxicity of biomaterials using liquid extracts allows us to determine whether the scaffold releases substances into the surrounding environment (cell culture medium) that reduce cell viability or whether it has the ability to absorb ingredients from the culture medium, which are necessary for cell growth (more information can be found in our previous publications^36,60^). In some cases, it may turn out that the extract obtained from the biomaterial does not show cytotoxic effect but its surface does not support cell adhesion, growth, proliferation, and differentiation.^61^ Therefore, it is also important to assess the response of cells seeded directly on the biomaterial. In our work, we started the estimation of the cell response in direct contact with the Curdlan-based scaffold by performing staining that allows the visualization of live and dead cells after 48 and 72 h of growth (using the commercial Live/Dead Double Staining Kit). Since we observed that both the “cartilage layer” and the “bone layer” of the biomaterial favored the growth of human chondrocytes and human osteoblasts, we additionally assessed cell proliferation quantitatively (by WST-8 assay) and qualitatively, i.e., by assessing cell morphology after staining the cell nuclei and cytoskeleton. These experiments confirmed that the Curdlan-based biomaterial not only favored cell colonization but also promoted their division over time. When considering the research of other scientists, it is observed that they also use both qualitative and quantitative methods to assess the cytocompatibility of osteochondral biomaterials. A good example is the work presented by Asensio et al.,^49^ which we mentioned in Section 3.1. The authors first indicated that an extract from an osteochondral scaffold did not exhibit the cytotoxic effect, and then they evaluated cytocompatibility in direct contact. Therefore, they seeded hAC and hOB cells on the biomaterial and after 1, 2, 7, and 14 days evaluated their number by assessing metabolic activity using the AlamarBlue assay. In addition, the authors estimated cell colonization after 14 days of incubation by staining cell nuclei (Hoechst 33342 dye) and free amines on the surface and inside the cells (Vybrant CFDA SE dye). The experiments showed that the number of cells growing on the biomaterial increased over time, and they colonized both the upper and lower parts of the scaffold. Another example includes the studies of Zhou et al.,^62^ who evaluated the cytocompatibility of 3D printed osteochondral biomaterials based on gelatin methacrylate and polyethylene glycol diacrylate in direct contact with human bone marrow-derived stem cells. The WST-8 test showed that the number of cells growing on the biomaterials was higher with an increasing incubation time (analysis was performed after 2, 4, and 6 days after cell seeding). Moreover, microscopic observations after staining cell nuclei (DAPI dye) and the cytoskeleton (Texas Red-X phalloidin dye) showed that the cells growing on the biomaterials were well flattened and possessed proper morphology. Therefore, these examples confirm that it is good practice to assess the cytocompatibility of an osteochondral scaffold not only quantitatively but also qualitatively to fully demonstrate that it supports the growth and proliferation of cultivated cells.

### 3.4 Inflammatory Response *In Vitro*

**Figure 4:**
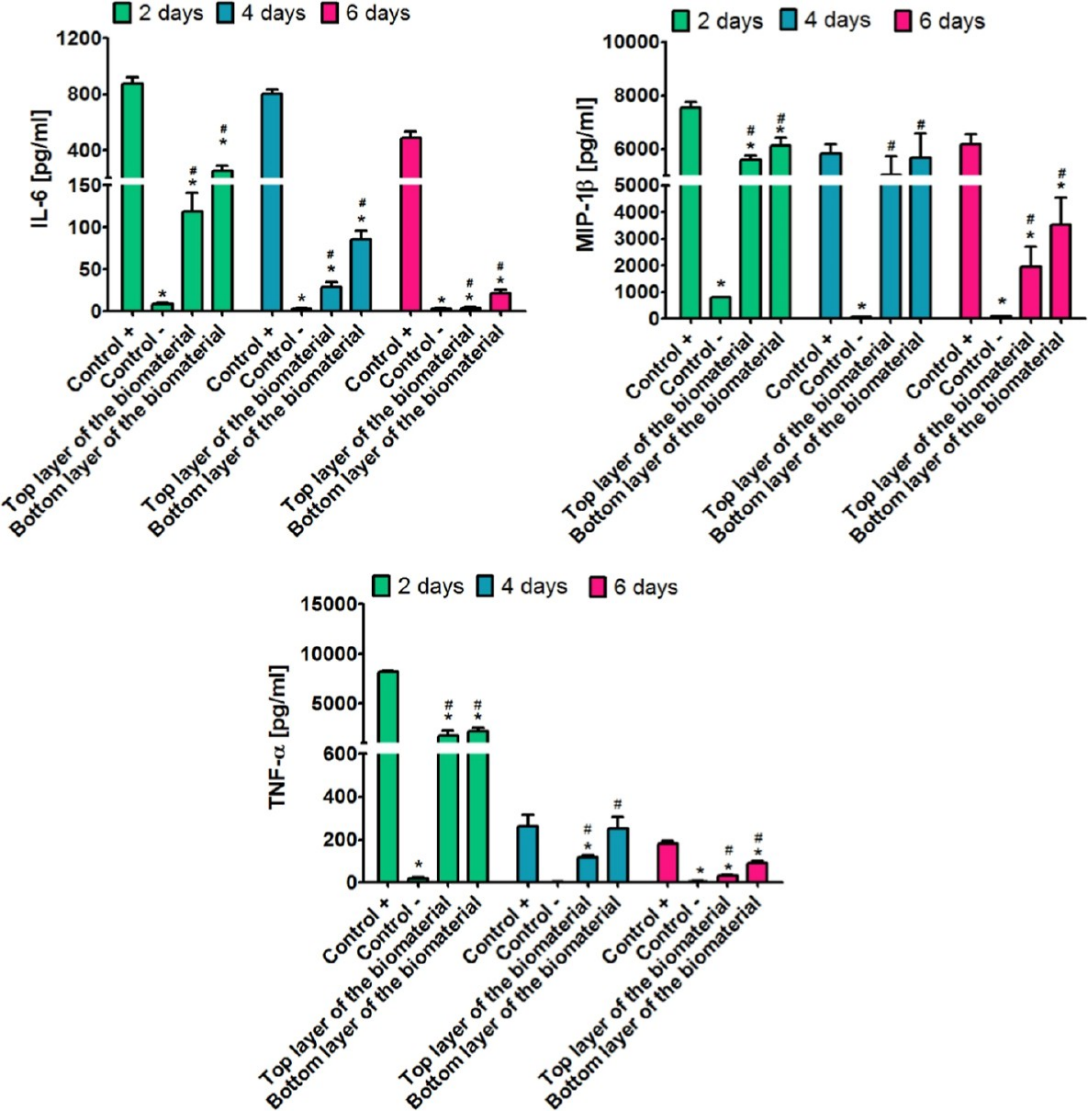
Inflammatory response of human macrophages (THP-1-derived cells) to the Curdlan-based biomaterial expressed by the amount of produced pro-inflammatory cytokines. Cells cultured on PS served as a negative control (Control−), while cells cultured on PS in the presence of 100 ng/mL LPS and 20 ng/mL INF-γ served as a positive control (Control+). * Statistically significant differences compared to the positive control (Control+), ^#^ Statistically significant differences compared to the negative control (Control−); (a two-way ANOVA test, followed by a Bonferroni comparison test, *P* < 0.05).

The implantation of a biomaterial is always associated with an inflammatory response to a foreign body. In this case, the main role is played by macrophages, which in direct contact with the biomaterial produce pro-inflammatory and anti-inflammatory cytokines. It is well known that short-term inflammation (i.e., production of pro-inflammatory cytokines by macrophages no longer than 3 weeks) is a beneficial phenomenon as it affects cell recruitment and the proper healing process. Nevertheless, prolonged inflammation associated with overproduction of pro-inflammatory cytokines by macrophages most often leads to implant failure.^39,63−65^ Therefore, the macrophage response to the Curdlan-based scaffold was also determined. The IL-6 ELISA test showed that THP-1-derived macrophages cultured in direct contact with both top and bottom layers of the Curdlan-based biomaterial produced the highest amount of IL-6 after 2 days of incubation (Figure 4). Although these amounts (approximately 120 pg/mL and 250 pg/mL) were significantly higher (*P* < 0.05) compared to the amount of IL-6 produced by macrophages cultured on PS (Control−, approximately 9 pg/mL), they were also significantly lower (*P* < 0.05) compared to the amount of this cytokine produced by macrophages grew on PS in the presence of LPS/INF-γ (Control+, approximately 870 pg/mL). With the increasing time of the experiment, it was observed that the amount of IL-6 produced by macrophages grew on biomaterials drastically decreased. Thus, on the sixth day of incubation, the amount of IL-6 produced by THP-1-derived macrophages was close to 4 pg/mL (cells cultured on the top layer of the biomaterial) and 20 pg/mL (cells cultured on the bottom layer of the biomaterial). The MIP-1β ELISA test (Figure 4) demonstrated that THP-1-derived macrophages cultured on top and bottom layers of the Curdlan-based biomaterial produced a comparable amount of this cytokine after both 2 and 4 days of incubation (Figure 4). On the sixth day of the experiment, a significant decrease in MIP-1β production by macrophages cultured on biomaterials was observed. Thus, the following amounts of MIP-1β were detected: approximately 90 pg/mL (cells cultured on PS, Control−), approximately 6000 pg/mL (cells cultured on PS in the presence of LPS/INF-γ, control+), approximately 1900 pg/mL (cells grew on the top layer of the biomaterial), and approximately 3500 pg/mL (cells grew on the bottom layer of the biomaterial). A similar trend was observed for the production of TNF-α to that for the production of IL-6 by macrophages (Figure 4). Indeed, after 2 days of incubation, the cells produced the greatest amount of TNF-α, and with increasing incubation time, these amounts decreased significantly. Thus, the amount of this cytokine produced by macrophages cultured on the top and bottom layers of the Curdlan-based biomaterial was approximately 1700 and 2100 pg/mL (after 2 days of incubation), approximately 115 and 250 pg/mL (after 4 days of incubation) as well as approximately 32 and 91 pg/mL (after 6 days of incubation), respectively. Therefore, the obtained results clearly showed that both the top and bottom layers of Curdlan-based biomaterials have the ability to induce short-term inflammation *in vitro* because the amount of all pro-inflammatory cytokines produced by macrophages decreased with increasing incubation time. Therefore, it seems that the Curdlan-based biomaterial should enable proper healing at the implantation site, without unfavorable prolonged inflammation.

Importantly, in our work, we assessed the response of macrophages in direct contact with the Curdlan-based biomaterial during a 6 day incubation. This allowed us to monitor the level of pro-inflammatory cytokines produced over time and observe a favorable downward trend. However, there are studies in which the *in vitro* response of macrophages in contact with osteochondral biomaterial is assessed only in the short term. For instance, Singh et al.^66^ and Joseph Christakiran et al.^67^ evaluated their silk-based osteochondral scaffolds only for 24 h. In these experiments, murine macrophages (RAW 264.7 cell line) were seeded on the wells of a PS plate, and then the biomaterials were placed on the cell layer. Similar to our study, the negative control consisted of cells growing in the culture medium only, while the positive control served cells growing in the culture medium supplemented with LPS. After that, the authors evaluated the level of produced TNF-α using the ELISA assay and observed that macrophages growing in contact with the osteochondral biomaterial produced a similar amount of this cytokine compared to negative control and at the same time significantly lower amount compared to the positive control. Based on these results, the authors concluded that silk-based biomaterials should not cause an unfavorable immune response and can be intended for implantation. Indeed, the conducted *in vivo* studies did not demonstrate any adverse inflammatory reaction after the implantation of these biomaterials. Nevertheless, in our opinion, a short-term assessment (e.g., during 24 h of incubation) of the inflammatory state *in vitro* will not always indicate clear conclusions. Therefore, a safer solution is to assess the immune response at several time points, taking into account the assessment of at least 2 pro-inflammatory cytokines, which allows for observing changes in cytokine’s amount and more precisely determining the fate of scaffold after implantation.

### 3.5 Analysis of the Responses to the Biomaterial *Ex Vivo*

**Figure 5:**
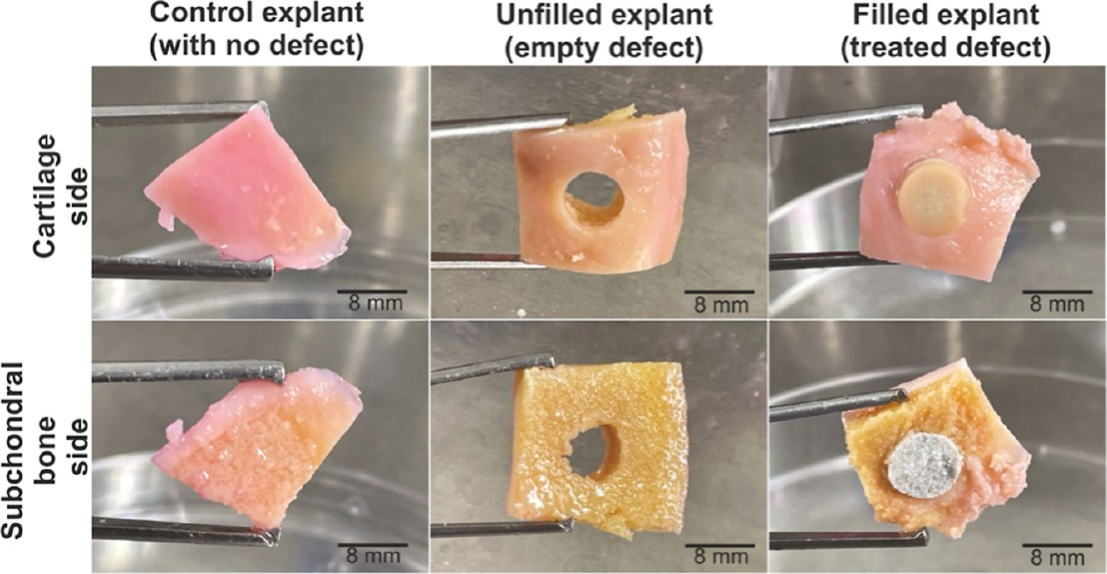
*Ex vivo* model of the human OC defect. Human osteoarthritic cartilage biopsies prepared as described in the Materials and Methods section were used as control conditions (control explant, without defect) or subjected to an experimental OC defect (8 mm punch)^40^ that was left empty (empty explant) or filled with the Curdlan-based OC biomaterial (filled explant) and were maintained for up to 14 days in the culture medium (scale bar = 8 mm, all representative data).

Following implantation, biomaterials generally interact with cells, surrounding tissues, and blood compounds with possible activation of detrimental responses against the implant. This complex process involves many types of cells, plasma proteins, and extracellular fluid components. In addition to assessing the impact of the biomaterial on such responses as initially tested toward human macrophages *in vitro* (Figure 4), it is critical to understand the behavior of the cells in the tissue surrounding the implant since an additional local inflammation may occur in this site after injury and potentially delay or even prevent proper healing processes.^63−65^ Furthermore, OC biomaterials are often being implanted in diseased tissues, e.g., those affected by osteoarthritis, where inflammation occurs at a very high extent. During such pathological conditions, both the chondrocytes in the articular cartilage and the osteoblasts in the subchondral bone may undergo phenotypic changes leading to the production of pro-inflammatory mediators, including IL-6, MIP-1β, and RANTES.^68^ Ideally, the implantation of a bioactive OC material should not trigger the secretion of such agents but rather reduce their expression levels.^69^ To assess the responses of chondrocytes and osteoblasts to the Curdlan-based OC biomaterial in an environment resembling the clinical situation, a study was performed by implanting it in an experimental *ex vivo* model of OC defect created in human osteoarthritic cartilage explant cultures^35^ (Figure 5).

**Figure 6:**
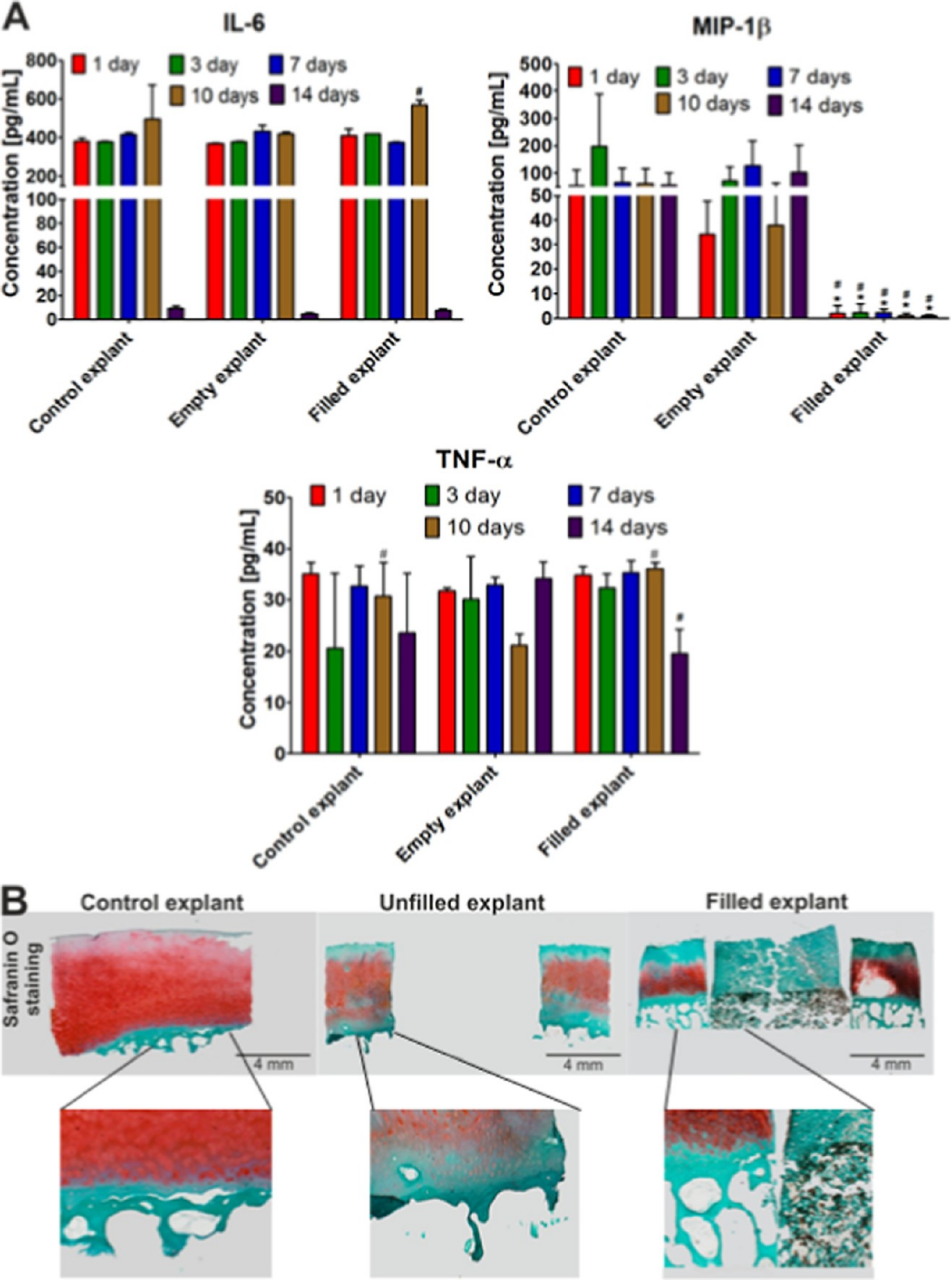
Analysis of the responses to the Curdlan-based OC biomaterial *ex vivo*. (A) Inflammatory responses in the various explant cultures (control explant, without defect; empty explant, i.e., OC defect left empty; filled explant, i.e., OC defect with the Curdlan-based OC biomaterial) were monitored by ELISA (IL-6, MIP-1β, and TNF-α) at the denoted time points for up to 14 days. * Statistically significant differences compared with the control explants (without defects) and ^#^ statistically significant differences compared with the empty explants (unfilled) (two-way ANOVA test, followed by a Bonferroni comparison test, *P* < 0.05). (B) Histological analysis in the various explant cultures was performed to detect cartilage matrix proteoglycans by safranin O staining after 14 days (magnification× 2, scale bar = 4 mm, with detailed views in the panels below, all representative data).

An evaluation of potential responses to the implantation of the Curdlan-based OC biomaterial by ELISA revealed that the amounts of IL-6 production in the control, unfilled, and filled explants remained at similar levels until day 10 of the analysis (approximately 400–600 pg/mL) (Figure 6A). However, a significant reduction of IL-6 production was observed in all the tested groups on day 14 (below 20 pg/mL, *P* < 0.05) (Figure 6A). Next, an estimation of the levels of MIP-1β by ELISA showed that the amounts of cytokine production in the explants filled with the Curdlan-based OC biomaterial were significantly lower than those noted in the control and unfilled explants throughout the whole period of analysis (14 days) (below 10 pg/mL versus approximately 100–400 and 40–200 pg/mL, respectively, *P* < 0.05) (Figure 6A). Finally, a measurement of the levels of TNF-α by ELISA demonstrated that the amounts of cytokine production were comparable between groups during 10 days, except for the unfilled explant at this particular time point (Figure 6A). However, a significant reduction of TNF-α production was seen in the explants filled with the Curdlan-based OC biomaterial on day 14 relative to the unfilled explants (approximately 20 pg/mL versus approximately 35 pg/mL, respectively, *P* < 0.05) (Figure 6A). These results reveal overall that the Curdlan-based OC biomaterial does not cause any unfavorable local IL-6 and TNF-α inflammation in the surrounding OC tissue while even promoting a beneficial local anti-inflammatory effect at the level of MIP-1β expression.

Another desirable feature of bioactive OC scaffolds is their compatibility with the adjacent OC tissue,^2,5,23,70,71^ the evaluation of which can also be performed in the above-mentioned experimental *ex vivo* model of OC defect.^72−75^ The various explants tested by ELISA were processed after 14 days of culture for an histological analysis of safranin O staining to assess the deposition of cartilage matrix proteoglycans and the adjustment of the Curdlan-based OC biomaterial with the surrounding cartilage (Figure 6B). The evaluation revealed the well-tolerated implantation of the biomaterial in the defects and its good compatibility with the subchondral bone layer after 14 days without any noticeable rejection (Figure 6B). Of note, bonding of the biomaterial with the surrounding cartilage tissue was not observed after 14 days, without perceptible deposition of matrix proteoglycans (Figure 6B). This might be due to the short time point of evaluation and/or to the absence of bone marrow arising from the subchondral bone during a clinical intervention like microfracture that would invade the defect and support the adhesion of the implant to the surrounding tissue. It should be emphasized that, in contrast to its top layer, the bottom layer of the biomaterial contains not only polymers (Curdlan and WPI) but also HAp granules known for their osteoinductive and osteoconductive properties and whose presence in polymer-based biomaterials accelerates osseointegration.^76−79^ It is therefore conceivable that the connection of the biomaterial with the cartilage tissue of the explants may occur at longer time points.

The development of an osteochondral biomaterial that simultaneously supports the regeneration of cartilage and subchondral bone, demonstrating complete integration with them, is still a challenge in modern medicine. To be able to initially assess the potential of a biomaterial to integrate with the surrounding tissues after implantation, it is worth conducting *ex vivo* studies using osteochondral explants.^73^ Here, we employed human explants where osteochondral defects were created using an 8 mm diameter punch for implantation of the current Curdlan-based biomaterial, mimicking standard surgeries in human patients. The analyses conducted allowed for an initial assessment of the biomaterial’s ability to integrate with the surrounding tissues (safranin O staining) and an evaluation of the inflammation state after implantation (estimation of the level of produced pro-inflammatory cytokines). These experiments allowed us to assume that the Curdlan-based biomaterial has the potential to integrate with the surrounding tissues after implantation without detrimental inflammatory reaction. It is worth emphasizing that most scientific papers currently present the results of *ex vivo* studies covering the implantation of osteochondral biomaterials in animal explants. Most often, these are porcine or bovine explants, but sometimes explants obtained from smaller animals such as rodents are used. Therefore, these explants enable the implantation of biomaterials with a significantly smaller diameter (approximately 4 mm) than in the case of biomaterials used in human medicine. Moreover, it is believed that osteochondral defects in small animals (although resembling defects in human in terms of their complexity) may show the ability to regenerate more rapidly and spontaneously.^75,80,81^ Therefore, during *ex vivo* studies of new biomaterials, it is worth using explants of human origin, especially since in the case of osteochondral explants, they are obtained during knee joint replacement (endoprosthetics), one of the most frequently performed orthopedic surgeries in the world.

### 3.6 Preliminary Toxicity Evaluation toward Zebrafish Larvae

**Figure 7:**
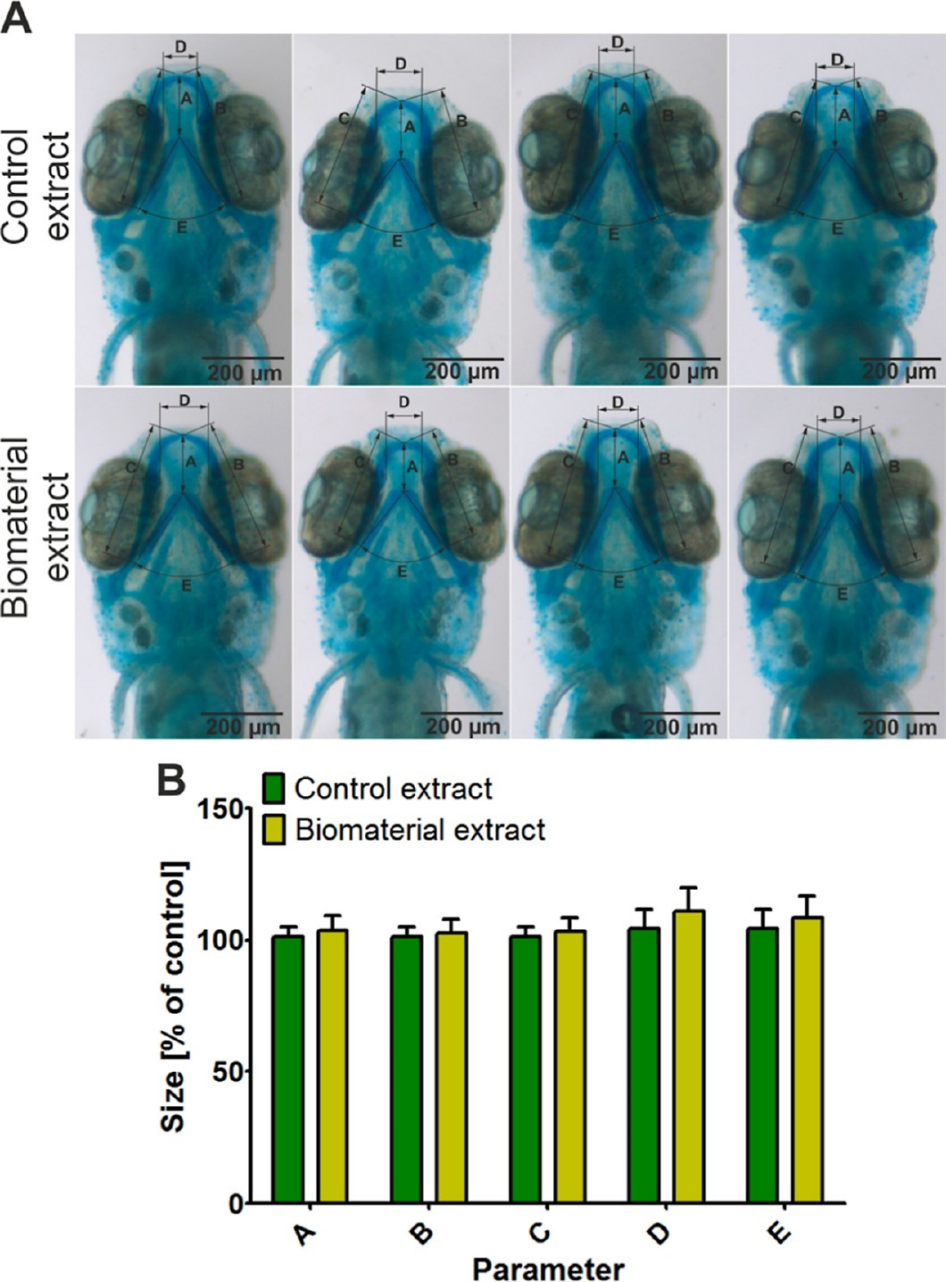
Alcian blue staining of cartilage in 7 dpf zebrafish larvae immersed in the extract from the Curdlan-based biomaterial or the control extract. Images were obtained under a Zeiss Axio Vert stereomicroscope, magnification 4×, scale bar = 200 μm (A). Cartilage development was assessed by measurements of the following parameters: distance between anterior Meckel’s cartilage and anterior ceratohyal (a), left anterior Meckel’s—posterior palatoquadrate length (b), right anterior Meckel’s—posterior palatoquadrate length (c), distance between anterior palatoquadrates (d), and ceratohyal angle (e), and measured parameters were presented as % of the control, *n* = 10; no significant differences in investigated parameters were observed between groups incubated with biomaterial extract and groups treated with control extract, *P* > 0.05 (B).

The *D. rerio* larvae model constitutes a great alternative compared to the mammalian model.^82,83^ This cost-effective animal model allows, among other things, the assessment of the toxicity impact of various substances on the development of the cartilage system. After incubation of zebrafish larvae in a medium containing toxic ingredients, the occurrence of craniofacial malformations such as deformed jaws, abnormal skull shape, or decrease in head trunk angle is observed.^84−87^ Since zebrafish model is also very often used for the compatibility assessment of biomaterials *in vivo*,^88−92^ in this study, a preliminary *in vivo* toxicity evaluation was performed by examining the effect of the biomaterial extract on the development of the craniofacial cartilage of *D. rerio* larvae. After 7 days of incubation, analysis of selected parameters characterizing cartilage development (Figure 7A) did not reveal any statistical changes between zebrafish larvae subjected to the extract from the Curdlan-based biomaterial and the control extract (Figure 7B), i.e., no malformations were observed (Figure 7A). Thus, these results showed that the biomaterial extract did not disturb the development of the craniofacial cartilage of *D. rerio*. It is therefore possible that it is nontoxic to cartilage *in vivo*.

As mentioned above, studies on the development of the cartilage system in the zebrafish larva model have primarily been used to investigate the toxicity of substances. Our study, for the first time, demonstrates that this model can also be used for the preliminary *in vivo* assessment of biomaterial’s toxicity.

### 3.7 Regeneration of the Amputated Caudal Fin in an Adult Zebrafish

**Figure 8:**
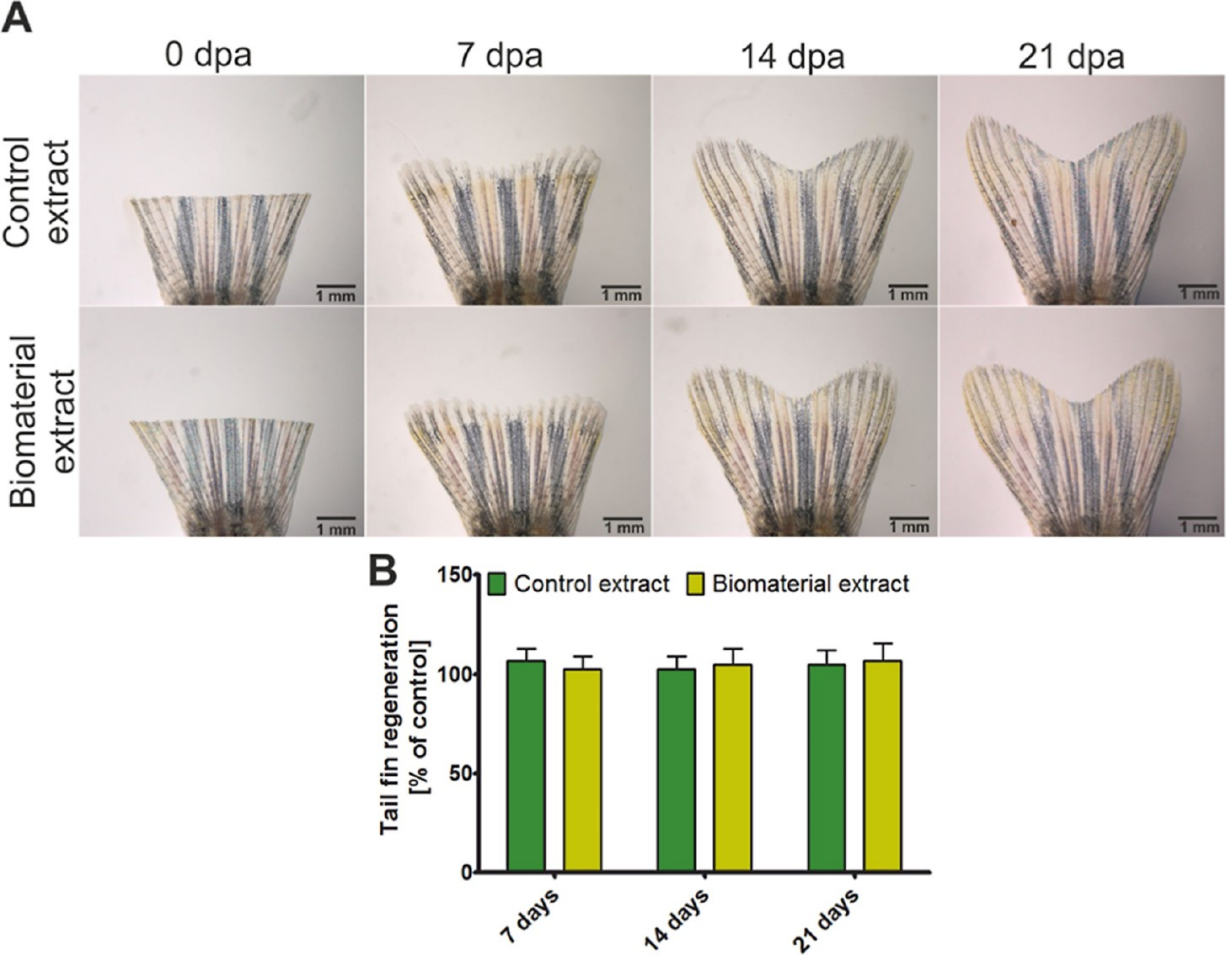
Caudal fin regrowth of adult zebrafish (3 month old) exposed to the extract from the Curdlan-based biomaterial or the control extract. Images obtained under a Zeiss Axio Vert stereomicroscope, magnification 1.6×, scale bar = 1 mm (A). Tail fin regeneration after 7, 14, and 21 days of amputation (dpa) was presented as% of the control, *n* = 10; no significant differences in tail fin regeneration were observed between groups incubated with the biomaterial extract and groups treated with the control extract, *P* > 0.05 (B).

*D. rerio* adults possess huge regenerative ability after partial amputation of its caudal fin and thanks to this, such model is very often used for the assessment of wound healing and the restoration of the bone tissue.^93−95^ During this complex process, osteoblasts play a crucial role, migrating to the site of damaged bones, proliferating and differentiating into mature cells, which enables bone remodeling, similar to those during a fracture in mammals.^93,94,96^ According to the available data, there are some substances that exhibit toxic activity, inhibiting the regrowth of the caudal fin,^97^ and there are others that may even stimulate this process.^98^ Therefore, in this experiment, the effect of the Curdlan-based biomaterial extract on the regeneration and regrowth of the amputated tail fin of *D. rerio* adults was investigated after 7, 14, and 21 days. Microscope observation (Figure 8A) showed no differences in caudal fin regrowth between adult fish immersed in the biomaterial extract and fish maintained in system water (control extract) at any observation time point (Figure 8B). These results showed that the Curdlan-based biomaterial did not reduce zebrafish caudal fin regeneration capacity, allowing for full regeneration of fish tail within approximately 21 days after amputation (dpa). Therefore, these data confirm the lack of toxicity of the Curdlan-based biomaterial *in vivo*.

Experiments assessing the regrowth of amputated caudal fins in adult zebrafish have so far been used primarily to study drugs, pharmaceuticals, and other active compounds. We have shown that they can also be used for the initial evaluation of the regenerative potential of osteochondral biomaterials. Both approaches, i.e., the assessment of cartilage system development in *D. rerio* larvae and the evaluation of caudal fin regrowth in adult zebrafish, provide valuable insights into the ability of osteochondral biomaterials to release neutral, beneficial, or harmful substances into the surrounding environment. These studies may be of particular importance in the case of biomaterials that are additionally enriched with medication.

### 3.8 Biomaterial Implantation in Veterinary Patients

Promising results of *in vitro*, *ex vivo*, and *in vivo* studies were the basis for the approval of the biomaterial for experimental therapy in 3 dogs suffering from lameness caused by OCD. The first veterinary patient—Tadzik (a 10 month-old St. Bernardyn)—had unfinished bone growth before the operation. Therefore, the surgery intervention was carried out within active growth zones (Figure S2A). Immediately after the operation, the dog’s lameness stopped. In X-ray after surgery (Figure S2B), the bottom layer of the biomaterial was clearly visible. In turn, the lack of shading by the top layer of the biomaterial may indicate that reconstruction of the articular surface will take place in the correct way. There was also a difference in the levels of the bony articular surface and the level of the implant, which may be due to the greater thickness of the top layer of the biomaterial compared to that of natural articular cartilage. Therefore, two reservations arised: 1) the subchondral bone canal prepared for implantation remained unfilled completely (in other words, the high of the resultant defect was greater than the high of the implanted Curdlan-based biomaterial). Such a phenomenon in mosaicplasty most often results in the formation of intraosseous cysts or causes migration of the graft into the prepared canal in the osteochondral tissue and 2) the prepared subchondral bone canal reached the growth cartilage, and as a consequence, it may cause disturbances in its activity and secondary bone deformation during further growth. One month after surgery, the X-ray image (Figure S2 C) showed the migration of the bottom layer of the biomaterial with a change in its shape and a decrease in its size. The biomaterial migrated to the metaphyseal zone, which may confirm the reservations in the image obtained immediately after the procedure and the fact of biodegradation of the biomaterial without a visible reactive bone reaction. However, the dog did not limp and was developing normally. In a clinical observation, no pain in the shoulder joint was noted. Three months after surgery, the X-ray image (Figure S2 D) showed further reduction of the biomaterial and a change in its shape. It also seems that the biomaterial migrated deeper into the subchondral bone canal. However, such an image may be caused by slightly different projections of the available photos. In the bone canal at the site of primary implantation of the graft, trabecularization is possible, as in natural bone. Nevertheless, this is only a guess. Importantly, the dog was not limping, and there was no pain in the clinical examination. The second veterinary patient, Ellie (a 1 year-old Border Collie), was already an adult female dog at the time of surgery. Therefore, the growth zones were not visible on the radiograph before the operation (Figure S3 A). On the other hand, on the edge of the glenoid, especially on the edge of the humeral head, an osteophytic bone superstructure was visible, which indicates the development of osteoarthritis. Taking into account the X-ray image taken immediately after implantation procedure (Figure S3 B), similar comments arised as in the case of first veterinary patient Tadzik (i.e., regarding the foundation of the biomaterial and the high of the prepared osteochondral canal). There was no pain in the clinical observations, but the lameness resolved completely 5 days after the implantation procedure. In the X-ray one month after the procedure (Figure S3 C), the degradation of the bottom layer of the Curdlan-based biomaterial was visible, and therefore, its dimensions were reduced. It also seems that the biomaterial migrated deeper along the osteochondral canal, but this migration was not as significant as that in the case of Tadzik. Ellie was not lame and showed no pain during clinical observations. Three months after surgery, further degradation of the bottom layer of the Curdlan-based biomaterial was observed. Ellie was not limping. A third veterinary patient named Bruno (a 6 month-old Bernese Mountain Dog) was in the midst of growing when he was brought to the veterinary clinic with symptoms of lameness. The necrosis focus was poorly visible on the radiograph taken before the procedure (Figure S4 A). The X-ray taken immediately after the operation (Figure S4 B) showed a good full filling of the osteochondral canal. The drilling did not exceed the boundaries of the growth plate. The fault on the articular surface between the environment and the biomaterial probably resulted from the difference in the thickness of the natural cartilage and the top later of the Curdlan-based biomaterial. The pain was noted after surgery, and lameness persisted. The lameness subsided only 14 days after the surgery, which was most likely associated with the perioperative pain; the lesion was very deep, and it was necessary to open shoulder joint wide. The X-ray image taken 1 month after the operation (Figure S4 C) showed complete degradation of the bottom layer of the biomaterial, with no visible bone reaction to its presence. Shadowing with an increased saturation with calcium salts was visible at the implantation site. The dog was not lame, and no pain was noted during clinical observations. Three months after the procedure, an almost complete closure of the growth zone of the humerus was observed on the radiograph (Figure S4 D). At the site of Curdlan-based biomaterial implantation, a moderate osseous translucence was visible. The bone structure visible in the area of radiolucency did not show signs of marginal sclerosis or abnormal weaving. On the medial side of the humeral neck had a fairly regular shadow surplus, which could correspond to the edges of the displaced bone component of the biomaterial. In turn, after 12 months of implantation (Figure S4 E), there was no excess shadow on the neck of the humerus, which was observed after 3 months. In the pace of the defect, there was a slight depression of the bony articular surface; below, there was visible brightening of the bone structure in the zone of the necrotic focus, but the trabecular scaffolding was restored. A band of bone sclerosis is visible at the border between healthy bone and the biomaterial.

From the interview conducted with the veterinarian performing the implantation procedures, it is known that all the three dogs do not limp and develop in a normal way. Thus, these experimental therapy results indicate that the Curdlan-based biomaterial is biocompatible *in vivo* and show that it may be considered as a promising candidate for treatment of OC defects in animals.

It is worth underlining that studies on veterinary patients may be considered equivalent for human clinical trials. Although the scientific literature describes cases of clinical studies using osteochondral scaffolds,^99−102^ to the best of our knowledge, there are no reports presenting cases of veterinary patients. Therefore, our studies show a new and interesting approach to the study of osteochondral biomaterials with the inclusion of veterinary patients.

## 4 Limitations

Although the current work shows a comprehensive panel of studies, including *in vitro*, *ex vivo*, and *in vivo* experiments, limitations of some of the analyses presented should be taken into account. First, it is necessary to mention *in vitro* assays according to the ISO standards. Although these are universal guidelines for biomaterials, they lack details regarding the specific type of the biomaterial. For this reason, they are interpreted differentially by scientists. Then, it should be emphasized that *ex vivo* research is conducted outside a living organism. Although numerous scientific works indicate that OC tissue explants can retain their viability and functions for up to several dozen days under *in vitro* conditions,^103−107^ they are not provided with biochemical and mechanical stimuli that naturally occur in the body. Therefore, one should take into account the fact that the lack of visible integration of the biomaterial with the surrounding tissue may be the result of the lack of external stimulation (including reparative and adhesive bone marrow elements) and not necessarily the properties of the biomaterial. The time of assessment may also be of significant importance. On the other hand, the assessment of inflammation in such conditions should also be treated as a preliminary assessment. Therefore, the *ex vivo* model should be considered as an intermediate model, i.e., constituting and experimental bridge between *in vitro* and *invivo* studies. In turn, *in vivo* tests on zebrafish include the assessment of the toxicity of the biomaterial, but only in indirect contact, i.e., using liquid extracts obtained from the scaffold. Therefore, these tests only allow us to determine whether the tested biomaterial releases harmful products into the environment or whether the released substances have pro-regenerative or antiregenerative properties or do not influence on the regenerative process. Nevertheless, according to the 3Rs principle, zebrafish is considered as an excellent model for preliminary evaluation of biomaterial’s toxicity *in vivo*. In turn, *in vivo* studies involving veterinary patients also have some drawbacks. The main problem is to obtain the appropriate number of patients (a statistically significant group, where the minimum number of patients is 3, *n* = 3) and the consent of their owners to carry out costly experimental therapy. Moreover, another problem with these types of experiments is the range of analyses performed to assess the integration of the biomaterial with the surrounding tissues. The most frequently performed evaluation is based on X-ray images because this method is relatively cost-effective and available in most veterinary clinics. However, such assessment sometimes gives ambiguous results (as shown in this study) and should be supported by more advanced techniques, such as magnetic resonance imaging (MRI) or computed tomography. However, these expensive analyses are usually not performed due to the refusal of the animal’s owners. This was also the case in our study. Research using veterinary patients may also involve difficulties in obtaining results in a timely manner. In our work, we planned to take X-rays before surgery and 1, 3, and 12 months after biomaterial’ implantation. Only in the case of one patient was it possible to obtain a complete set of X-ray images. Nevertheless, these studies constitute the highest quality evidence of the effectiveness of the implanted biomaterial, as observation can be carried out throughout the patient’s life (of course, if there is contact with it). To summarize, it is worth emphasizing that each study has its advantages and disadvantages and that no ideal models for biological studies have been developed so far. Nevertheless, we believe that the presented set of studies provides valuable knowledge about osteochondral biomaterials, the value of which exceeds all of the limitations mentioned above.

## 5 Conclusions

The growing demand for biomaterials among society and the development of materials engineering have resulted in the fabrication of new biomaterials each year, which consist of both natural and synthetic polymers and are produced using increasingly modern production methods.^108−112^ However, in order for a new biomaterial to be recognized as a promising candidate for use in regenerative medicine, it must undergo a series of tests confirming its usefulness and biological safety. In the case of biological research, *in vivo* experiments involving laboratory animals are an essential element confirming the biocompatibility of a biomaterial. However, there is a trend in accordance with the 3Rs principle, which indicates that the number of biological studies involving laboratory animals should be limited to a minimum. For this reason, there is a justified need to develop alternative models (sets of experiments) that will allow for the selection of the most promising variants of biomaterials before they are intended for *in vivo* research involving laboratory animals.^61,113,114^ In this study, a modern approach to testing the biocompatibility of OC scaffolds in accordance with the 3Rs principle was presented. A biphasic Curdlan-based scaffold as a model biomaterial was subjected to comprehensive biological evaluations *in vitro*, *ex vivo*, and *in vivo*. *In vitro* experiments showed that the biomaterial did not exhibit any cytotoxicity, mutagenicity, and genotoxicity. Additionally, it was noted that the biomaterial supported the viability and proliferation of human chondrocytes and osteoblasts and had the ability to induce short-term inflammation in contact with human macrophages. *Ex vivo* experiments demonstrated that the selected biomaterial did not induce a local inflammation in the surrounding OC tissue with which it had good compatibility without any sign of rejection after 14 days of incubation. *In vivo* studies in the zebrafish model showed that the biomaterial was not toxic, enabling proper development of the cartilage system in larvae and an adapted regeneration of the caudal fin in adults. In turn, X-ray observations demonstrated that the Curdlan-based biomaterial promoted tissue regeneration following implantation in OC defects in three dogs (veterinary patients) suffering from OCD. Thus, it was demonstrated that the biphasic OC biomaterial based on Curdlan is characterized by biological safety, which confirms the aim of this study. This set of advanced biological tests allows for the assessment of the biomedical potential of new OC scaffolds and is a useful tool for other scientists developing these types of biomaterials. Importantly, this study is in line with current research trends as it will allow for reducing the use of laboratory animals. To the best of our knowledge, there are no papers in the currently available literature that present a set of biological studies enabling the assessment of the biomedical potential of osteochondral scaffolds, taking into account the 3Rs principle, which gives the innovative character of this work. Moreover, some of the presented analyses were applied for the first time to evaluation of osteochondral biomaterials (e.g., studies on zebrafish). This set of experiments proposed by us is not limited to only one type of studies (e.g., at the *in vitro* level) but combines studies at different levels of advancement (*invitro*, *ex vivo*, and *in vivo*), which allows their use together (as a package of experiments) but also provides a range of methods that can be used as a complement to other experiments.

## Supporting Information Available

Intraoperative photos showing the implantation procedure of Curdlan-based biomaterial; X-ray images of a 10 month-old St. Bernard named Tadzik; X-ray images of a 1 year-old Border Collie named Ellie; X-ray images of a 6 month-old Bernese mountain dog named Bruno; examples of microscope images showing results obtained in chromosome aberration assay *in vitro*; examples of microscope images showing results obtained in micronucleus (MNvit) assay *in vitro*; and examples of microscope images showing results obtained in Comet assay *in vitro* (PDF)

## Notes

Patents: The Curdlan-based scaffold was fabricated according to the Polish patent no. 240639 “Biphasic biomaterial based on Curdlan and hydroxyapatite (HAp) for regeneration of osteochondral defects and the method of its preparation”.

## Abbreviations

3Rs: replacement, reduction, refinement
ATCC: American Type Culture Collection
BN: binucleated cells
BNMN: binucleated cells with micronuclei
CBPI: cytokinesis-block proliferation index
CHO-K1: Chinese hamster cells
CLSM: confocal laser scanning microscope
DMSO: dimethyl sulfoxide
FBS: fetal bovine serum
HAp: hydroxyapatite
hChon: human chondrocytes
hFGF-2: human fibroblast growth factor 2
hFOB 1.19: human fetal osteoblasts
hTGF-β1: human transforming growth factor β-1
IFN-γ: interferon gamma
IL-6: interleukin 6
LPS: lipopolysaccharide
MIP-1β: macrophage inflammatory protein 1 beta
MN: micronuclei
OC: osteochondral
OCD: *Osteochondritis Dissecans*
PMA: phorbol 12-myristate 13-acetate
PS: polystyrene
S9: cofactor-supplemented post mitochondrial factor
THP-1: human acute monocytic leukemia cells
TNF-α: tumor necrosis factor alpha
WPI: whey protein isolate
