# Supplementary material for: Modern Approach to Testing the Biocompatibility of Osteochondral Scaffolds in Accordance with the 3Rs Principle—Preclinical In Vitro, Ex Vivo, and In Vivo Studies Using the Biphasic Curdlan-Based Biomaterial
Source: ACS Biomater Sci Eng. 2025 Jan 20;11(2):845–65. doi: 10.1021/acsbiomaterials.4c01107 (PMC11815629; doi:10.1021/acsbiomaterials.4c01107)
Supplement: Supplementary file 1 — ab4c01107_si_001.pdf [file ab4c01107_si_001.pdf]

Supporting Information

A modern approach to testing the biocompatibility of osteochondral scaffolds in accordance with the 3Rs principle – Preclinical *in vitro*, *ex vivo*, and *in vivo* studies using biphasic curdlan-based biomaterial

Katarzyna Klimek[a,*], Sylwia Terpilowska[b], Agnieszka Michalak[c], Rafal Bernacki[d], Aleksandra Nurzynska[a], Magali Cucchiarini[e], Marta Tarczynska[f], Krzysztof Gaweda[f], Stanisław Głuszek[b], Grazyna Ginalska[a,g]

[a]Medical University of Lublin, Chair and Department of Biochemistry and Biotechnology, Chodzki 1 Street, 20-093 Lublin, Poland

[b]Jan Kochanowski University, Collegium Medicum, Department of Surgical Medicine with the Laboratory of Medical Genetics, IX Wiekow Kielc 19A Av., 25-317 Kielce, Poland

[c]Medical University of Lublin, Independent Laboratory of Behavioral Studies, Chodzki 4a Street, 20-093 Lublin, Poland

[d]Veterinary Clinic Aura, Debowa 31 Street, 86-065 Lochowo, Poland

[e]Center of Experimental Orthopaedics, Saarland University Medical Center, Saarland University, Kirrbergerstr. Bldg 37, 66421 Homburg/Saar, Germany

[f]Medical University of Lublin, Department and Clinic of Orthopaedics and Traumatology, Jaczewskiego 8 Street, 20-954 Lublin, Poland

[g]Vincent Pol University, Faculty of Health Sciences, Choiny 2 Street, 20-816 Lublin, Poland

Corresponding author:

*Dr. Katarzyna Klimek; E-mail: katarzyna.klimek@umlub.pl

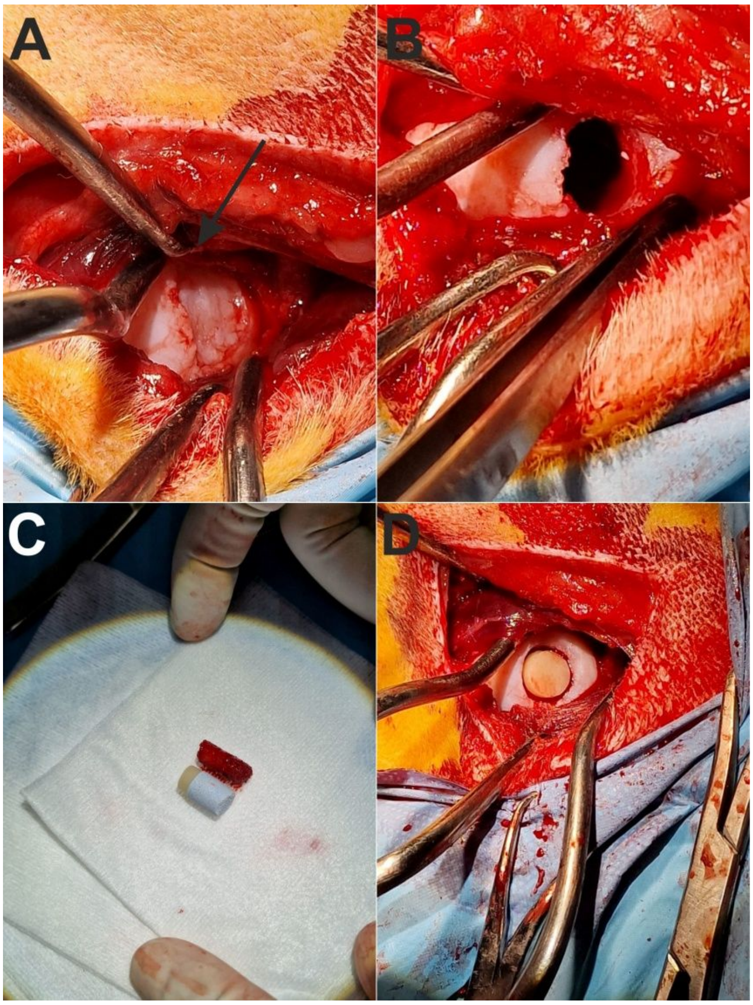

Fig. S1. Intraoperative photos showing the implantation procedure of curdlan-based biomaterial. Damaged cartilage (A); Removal of damaged cartilage with a fragment of subchondral bone (B); Adjustment of the biomaterial to the osteochondral plug (C); Placement of the biomaterial in the drilled osteochondral canal (D).

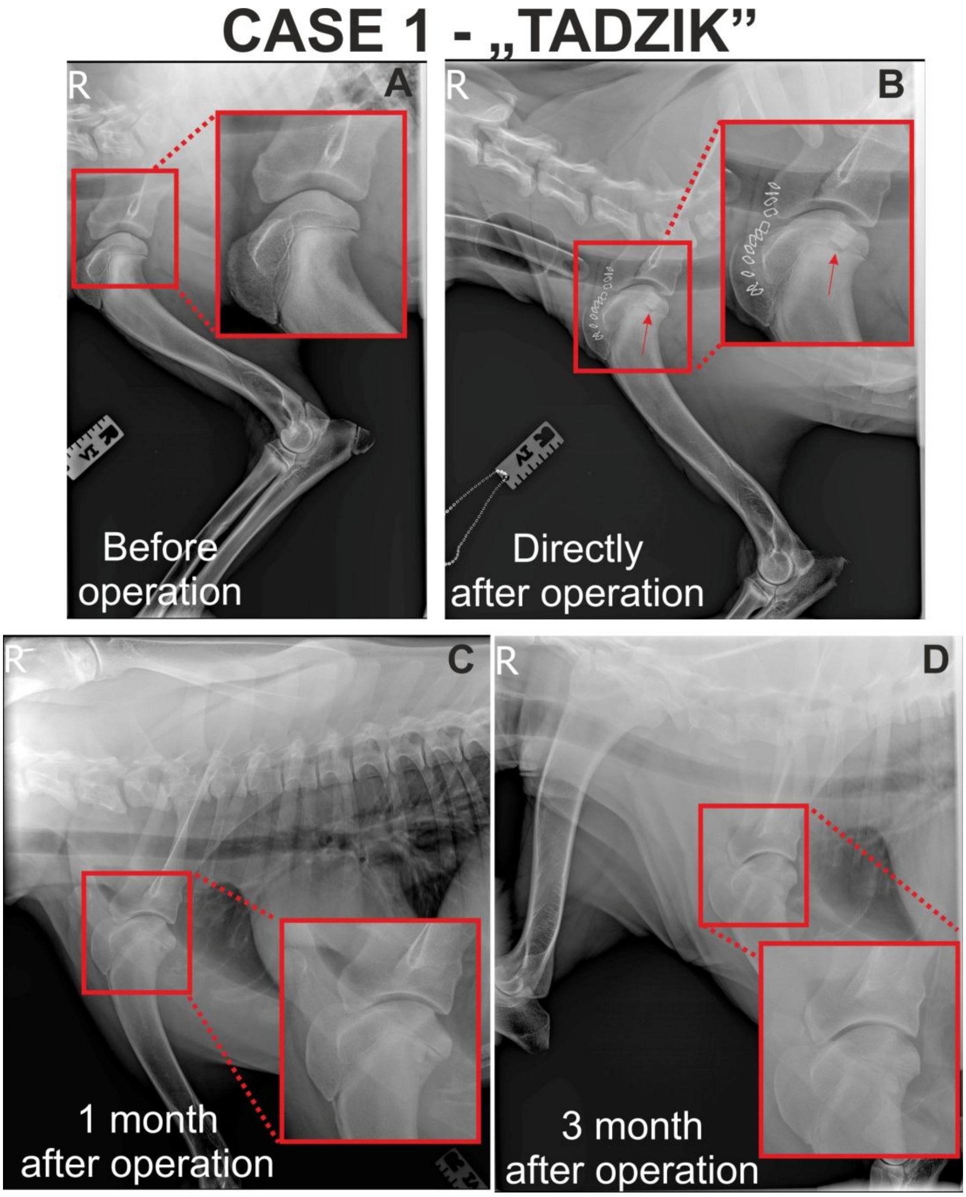

Fig. S2. X-ray images of a 10-month-old St. Bernard named Tadzik. Radiographs were taken before the implantation procedure (A), immediately after the procedure (B), 1 month after the procedure (C), and 3 months after the procedure (D).

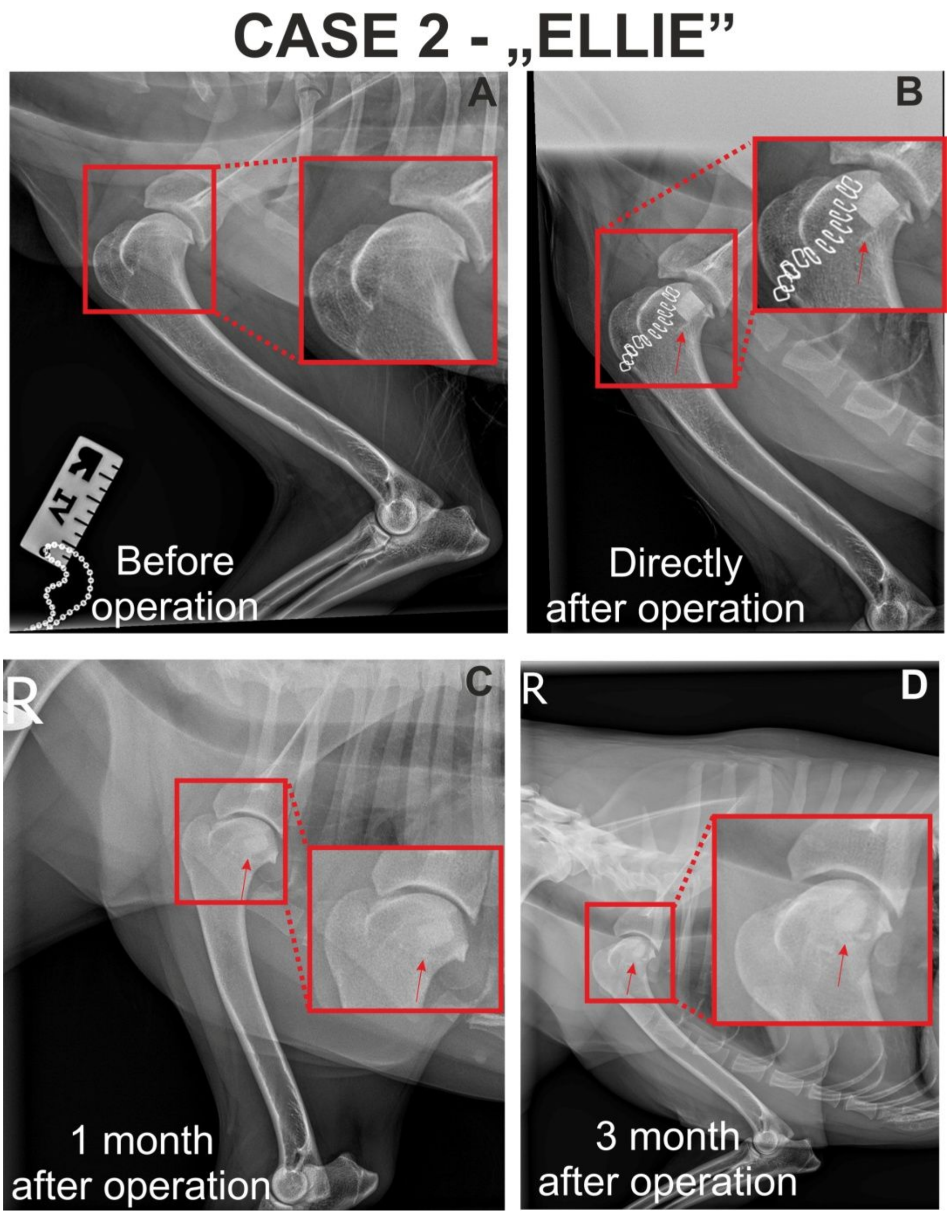

Fig. S3. X-ray images of a 1-year-old Border Collie named Ellie. Radiographs were taken before the implantation procedure (A), immediately after the procedure (B), 1 month after the procedure (C), and 3 months after the procedure (D).

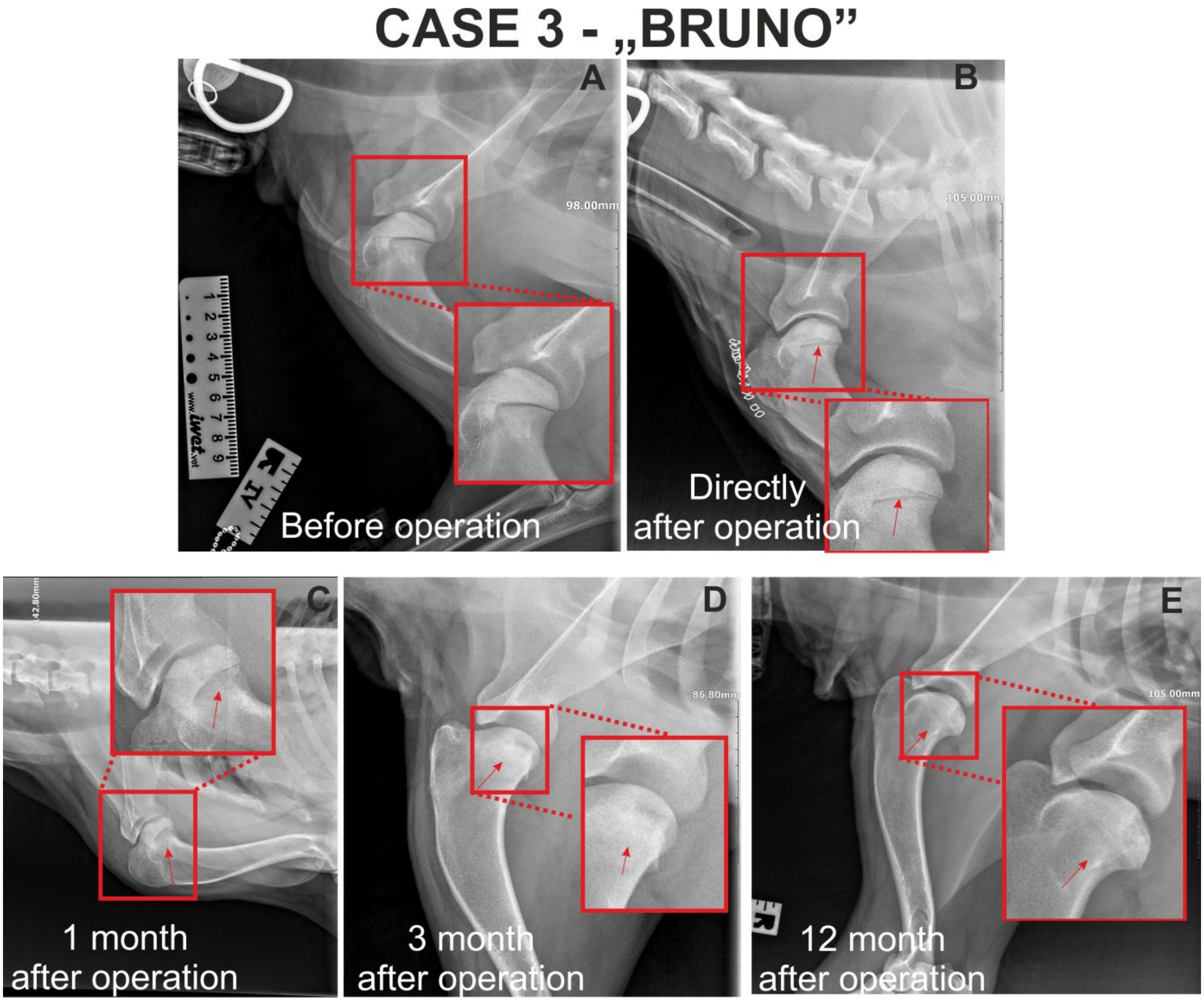

Fig. S4. X-ray images of a 6-month-old Bernese mountain dog named Bruno. Radiographs were taken before the implantation procedure (A), immediately after the procedure (B), 1 month after the procedure (C), 3 months after the procedure (D) and 12 month after the procedure (E).

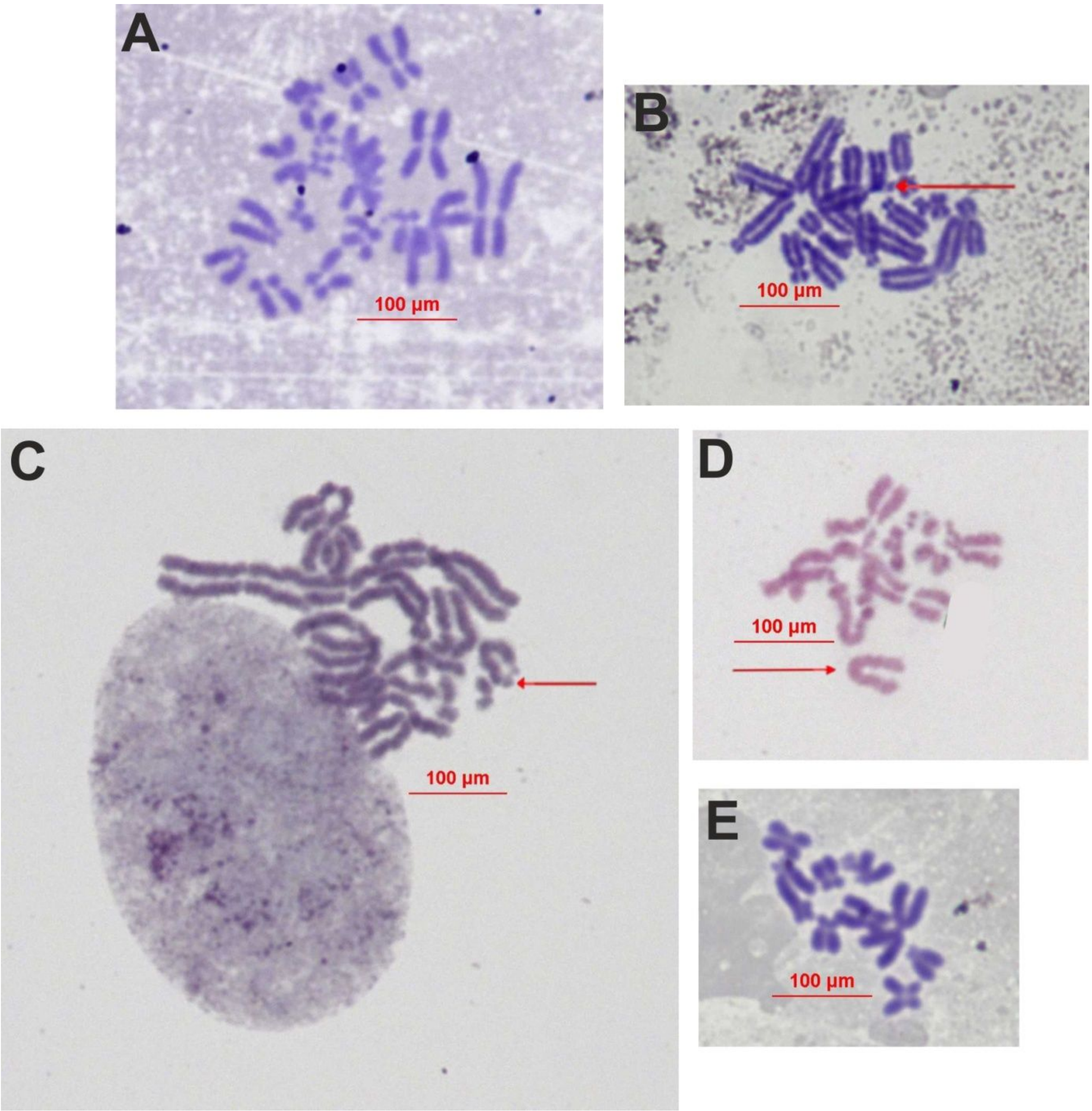

Fig. S5. Examples of microscope images showing results obtained in chromosome aberration assay *in vitro*. Normal chromosomes (A) were observed after treatment of CHO-K1 cells with control extracts and curdlan-based biomaterial extracts. Chromatid breaks (B), fragments, quadriradical (C), deletion (D), and losses of chromosomes (E) was noted after exposure of CHO-K1 cells with positive reference substances. Structural changes were observed after staining the CHO-K1 cells with 0.7% Giemsa solution.

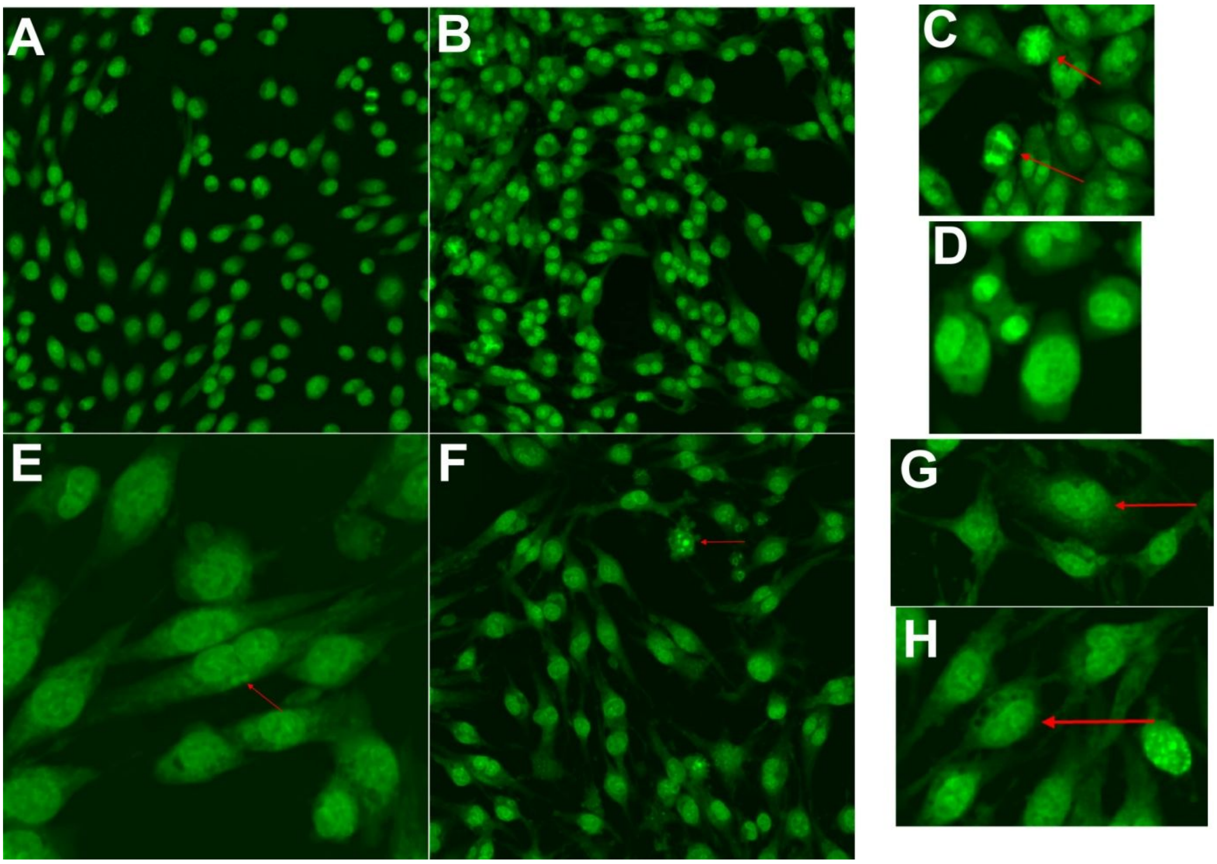

Fig. S6. Examples of microscope images showing results obtained in micronucleus (MNvit) assay *in vitro*. Normal, dividing cells were observed after treatment of CHO-K1 cells with control extracts (A) and curdlan-based biomaterial extracts (B). Thus, cells in anaphase (C), telophase (D), and metaphase (C) were present. In turn, micronuclei formation (Fig. E), displayed characteristic apoptosis in cells (F), nuclear convulsion, fragmentation, cytoplasmic blebbing, giant cells, and cytoplasmic vacuolation (G, H) were noted in CHO-K1 cells incubated with positive reference substances. The CHO-K1 cells were observed after staining with acridine orange solution.

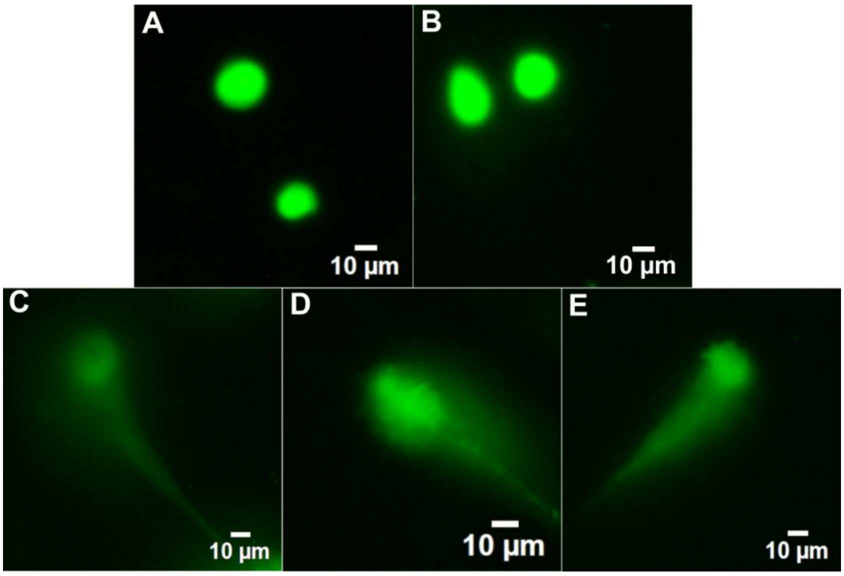

Fig. S7. Examples of microscope images showing results obtained in Comet assay *in vitro*. Normal cells without comet formation, namely without presence of deoxyribonucleic acid (DNA) tail were observed after treatment of CHO-K1 cells with control extracts (A) and curdlan-based biomaterial extracts (B). In turn, the increase in DNA damage (i.e. DNA tail presence) was observed in cells incubated with all positive reference substances (C-E).
